# Characteristics, Outcomes, and Severity Risk Factors Associated With SARS-CoV-2 Infection Among Children in the US National COVID Cohort Collaborative

**DOI:** 10.1001/jamanetworkopen.2021.43151

**Published:** 2022-02-08

**Authors:** Blake Martin, Peter E. DeWitt, Seth Russell, Adit Anand, Katie R. Bradwell, Carolyn Bremer, Davera Gabriel, Andrew T. Girvin, Janos G. Hajagos, Julie A. McMurry, Andrew J. Neumann, Emily R. Pfaff, Anita Walden, Jacob T. Wooldridge, Yun Jae Yoo, Joel Saltz, Ken R. Gersing, Christopher G. Chute, Melissa A. Haendel, Richard Moffitt, Tellen D. Bennett

**Affiliations:** 1 Section of Critical Care Medicine, Department of Pediatrics, University of Colorado School of Medicine, University of Colorado, Aurora; 2 Section of Informatics and Data Science, Department of Pediatrics, University of Colorado School of Medicine, University of Colorado, Aurora; 3 Department of Biomedical Informatics, Stony Brook University, Stony Brook, New York; 4 Palantir Technologies, Denver, Colorado; 5 Johns Hopkins University School of Medicine, Baltimore, Maryland; 6 Translational and Integrative Sciences Center, University of Colorado, Aurora; 7 Center for Health AI, University of Colorado, Aurora; 8 North Carolina Translational and Clinical Sciences Institute), University of North Carolina at Chapel Hill, Chapel Hill; 9 National Center for Advancing Translational Sciences, National Institutes of Health, Bethesda, Maryland; 10 Schools of Public Health, and Nursing, Johns Hopkins University, Baltimore, Maryland

## Abstract

**Importance:**

Understanding of SARS-CoV-2 infection in US children has been limited by the lack of large, multicenter studies with granular data.

**Objective:**

To examine the characteristics, changes over time, outcomes, and severity risk factors of children with SARS-CoV-2 within the National COVID Cohort Collaborative (N3C).

**Design, Setting, and Participants:**

A prospective cohort study of encounters with end dates before September 24, 2021, was conducted at 56 N3C facilities throughout the US. Participants included children younger than 19 years at initial SARS-CoV-2 testing.

**Main Outcomes and Measures:**

Case incidence and severity over time, demographic and comorbidity severity risk factors, vital sign and laboratory trajectories, clinical outcomes, and acute COVID-19 vs multisystem inflammatory syndrome in children (MIS-C), and Delta vs pre-Delta variant differences for children with SARS-CoV-2.

**Results:**

A total of 1 068 410 children were tested for SARS-CoV-2 and 167 262 test results (15.6%) were positive (82 882 [49.6%] girls; median age, 11.9 [IQR, 6.0-16.1] years). Among the 10 245 children (6.1%) who were hospitalized, 1423 (13.9%) met the criteria for severe disease: mechanical ventilation (796 [7.8%]), vasopressor-inotropic support (868 [8.5%]), extracorporeal membrane oxygenation (42 [0.4%]), or death (131 [1.3%]). Male sex (odds ratio [OR], 1.37; 95% CI, 1.21-1.56), Black/African American race (OR, 1.25; 95% CI, 1.06-1.47), obesity (OR, 1.19; 95% CI, 1.01-1.41), and several pediatric complex chronic condition (PCCC) subcategories were associated with higher severity disease. Vital signs and many laboratory test values from the day of admission were predictive of peak disease severity. Variables associated with increased odds for MIS-C vs acute COVID-19 included male sex (OR, 1.59; 95% CI, 1.33-1.90), Black/African American race (OR, 1.44; 95% CI, 1.17-1.77), younger than 12 years (OR, 1.81; 95% CI, 1.51-2.18), obesity (OR, 1.76; 95% CI, 1.40-2.22), and not having a pediatric complex chronic condition (OR, 0.72; 95% CI, 0.65-0.80). The children with MIS-C had a more inflammatory laboratory profile and severe clinical phenotype, with higher rates of invasive ventilation (117 of 707 [16.5%] vs 514 of 8241 [6.2%]; *P* < .001) and need for vasoactive-inotropic support (191 of 707 [27.0%] vs 426 of 8241 [5.2%]; *P* < .001) compared with those who had acute COVID-19. Comparing children during the Delta vs pre-Delta eras, there was no significant change in hospitalization rate (1738 [6.0%] vs 8507 [6.2%]; *P* = .18) and lower odds for severe disease (179 [10.3%] vs 1242 [14.6%]) (decreased by a factor of 0.67; 95% CI, 0.57-0.79; *P* < .001).

**Conclusions and Relevance:**

In this cohort study of US children with SARS-CoV-2, there were observed differences in demographic characteristics, preexisting comorbidities, and initial vital sign and laboratory values between severity subgroups. Taken together, these results suggest that early identification of children likely to progress to severe disease could be achieved using readily available data elements from the day of admission. Further work is needed to translate this knowledge into improved outcomes.

## Key Points

**Question:**

What are the characteristics, changes over time, outcomes, and severity risk factors of children with SARS-CoV-2 within the National COVID Cohort Collaborative?

**Findings:**

In this cohort study, 167 262 children at 56 sites were SARS-CoV-2–positive and 10 245 were hospitalized. Several demographic and comorbidity variables and many initial vital sign and laboratory test values were associated with higher peak illness severity.

**Meaning:**

This study noted clinical data elements that could assist with early identification of children at risk for severe disease due to SARS-CoV-2 infection.

## Teaser

This cohort study examines factors that may have utility in estimating severity and outcomes of children with SARS-CoV-2 infection.

## Introduction

As of January 2022, SARS-CoV-2 had infected more than 290 million people and caused more than 5.4 million deaths worldwide.^1^ The associated infection, COVID-19, is characterized by pneumonia, hypoxemic respiratory failure, cardiac and kidney dysfunction, and substantial mortality and morbidity. Although children often experience milder illness,^2,3,4^ SARS-CoV-2 can cause severe pediatric disease via both acute COVID-19 and multisystem inflammatory syndrome in children (MIS-C).^5,6,7,8,9,10^ MIS-C is a hyperinflammatory, postinfectious complication of SARS-CoV-2.^5,11,12^ Characterized by cardiovascular, respiratory, neurologic, gastrointestinal, and mucocutaneous manifestations and organ dysfunction, more than 5900 cases of MIS-C have been reported to the US Centers for Disease Control and Prevention, with more than half of the patients requiring intensive care unit admission and greater than one-third experiencing shock.^13,14^

Research in pediatric COVID-19 and MIS-C has been slowed by the lack of large, multi-institutional data sets of affected children. Investigators in Europe^7^ and the US^8,13,15^ have reported multicenter studies, but analysis of individual patient vital sign and laboratory data was absent. An extensive, granular, representative clinical data set is needed to improve our understanding of the presentation, risk factors, and severity signals of pediatric COVID-19 and MIS-C.

The National COVID Cohort Collaborative (N3C) was formed to improve understanding of SARS-CoV-2 via a novel approach to data sharing and analytics.^16^ The N3C is composed of members from the National Institutes of Health Clinical and Translational Science Awards Program, its Center for Data to Health, the IDeA Centers for Translational Research,^17^ and several electronic health record–based research networks.^18^ N3C structure, data ingestion/integration, supported common data models, and patient variables have been previously described^16,18^ (eMethods in the Supplement provides N3C patient inclusion criteria).

Our objective was to provide a detailed clinical characterization of the largest cohort of US pediatric SARS-CoV-2 cases to date. We hypothesized we could (1) identify risk factors for higher severity disease among hospitalized children, (2) describe SARS-CoV-2 case and hospitalization rates over time, (3) visualize changes in SARS-CoV-2 medication treatment regimens over time, (4) compare changes in vital sign and laboratory results between hospitalized children with varying clinical severity, and (5) identify differences in risk factors and outcomes between children with acute COVID-19 vs MIS-C. In addition, at the time of the study, Centers for Disease Control and Prevention reporting indicated the Delta (B.1.617.2) variant was the predominant SARS-CoV-2 strain, surpassing 50% of US specimens tested during the 2-week period ending June 26, 2021.^19^ Given reports of increased transmissibility^20^ and hospitalization among adults^21^ and children^22^ with SARS-CoV-2 infection, we also report preliminary data comparing demographic characteristics, comorbidities, and clinical outcomes of infected children with SARS-CoV-2 infection in the pre-Delta era (before June 26, 2021) with the Delta era (beginning June 26, 2021).

## Methods

### Cohort Definition

We performed an analysis of all children younger than 19 years at the first SARS-CoV-2 testing at the 56 N3C sites if their index encounter (eMethods in the Supplement) ended before September 24, 2021. Children were considered to have SARS-CoV-2 if they had a positive SARS-CoV-2 polymerase chain reaction (PCR), antigen (Ag), or antibody test result. Children were considered to have MIS-C if they were hospitalized with a positive SARS-CoV-2 test result and assigned either of 2 recommended *International Statistical Classification of Diseases, 10th Revision* diagnosis codes for MIS-C: during the early phase of the pandemic, M35.8,^23^ or the more specific code M35.81 introduced October 1, 2020, and effective January 1, 2021.^24^ The N3C Data Enclave is approved under the authority of the National Institutes of Health Institutional Review Board. Each N3C site maintains an institutional review board–approved data transfer agreement. The analyses in this article were approved by institutional review boards from each institution for study investigators with data access (eMethods in the Supplement), including the waiver of informed consent. Study results are reported in accordance with the Strengthening the Reporting of Observational Studies in Epidemiology (STROBE) reporting guideline for cohort studies.^25^

For children with positive SARS-CoV-2 test results within the N3C, we describe the geographic location, monthly incidence, and changes in pediatric age distribution and maximum clinical severity over time. We determined the proportion of hospitalized children given different antimicrobial and immunomodulatory medications and provide corresponding adult changes over time for comparison. We compared the clinical characteristics, outcomes, and laboratory test profiles of children with MIS-C with those of children with acute COVID-19 (positive SARS-CoV-2 PCR/Ag test but no MIS-C diagnosis code or positive antibody result). In addition, we report preliminary results comparing the demographic characteristics, comorbidities, and outcomes of children infected in the Delta era (encounter start date after June 26, 2021) with the pre-Delta era.

### Hospital Index Encounter and Clinical Severity Definition

For each child with laboratory-confirmed SARS-CoV-2, we selected the associated encounter demonstrating the maximum Clinical Progression Scale score created by the World Health Organization for COVID-19 clinical research^18,26^ (eMethods in the Supplement). Clinical severity categories include mild (outpatient or emergency department visits only), moderate (hospitalized), and severe (hospitalized and requiring invasive ventilation, vasopressor-inotropic support, or extracorporeal membrane oxygenation, or death). eMethods in the Supplement provides additional information.

### Statistical Analysis

For each clinical concept (eg, laboratory measure, vital sign, medication, or comorbidity) we defined or identified existing concept sets in the Observational Medical Outcomes Partnership^27^ common data model. We thus identified children with the preselected comorbidities of asthma, diabetes types 1 and 2, and obesity, given reports of severe disease among such patients.^8,9^ We identified children with chronic medical conditions using the pediatric complex chronic conditions (PCCC) algorithm via adaptation of prior use of R software implementation.^28,29^

We assessed the outcomes associated with potential clinical severity risk factors (eg, demographic or comorbidity variable) by calculating the odds of severe vs moderate peak clinical severity using multivariable logistic regression. All patient demographic characteristics were obtained from each site’s electronic health record database. Race and ethnicity data provided by N3C sites were also analyzed to assist with identification of patients at higher risk of severe disease. Classifications for race and ethnicity are made using the data provided to the N3C from each health care site. How an individual's race and ethnicity are determined and then stored in their electronic health record is at the discretion of each health care site. We used χ^2^ deviance tests to evaluate changes in relative proportions of predefined pediatric age ranges over time and changes in proportions of moderate and severe cases over time. We evaluated differences in initial vital sign and laboratory values between moderate and severe subgroups using generalized estimating equations with an exchangeable working correlation structure. We assessed the change over time for each vital sign or laboratory test during a patient’s hospitalization using a linear model to average over the first week of values. We used these model results to estimate the difference between hospital day 0 and day 7 for each severity subgroup. In addition, we determined the change in odds of severe vs moderate disease in the pre-Delta era with the Delta era (overall and for each preselected risk factor), using multivariable logistic regression. As a sensitivity analysis, we determined the association between health care site and the strength of each variable’s association with severe disease, using a 2-sided significance threshold of *P* < .05. Study analyses were performed using Foundry Code Workbook, version 4.339.0 (Palantir Technologies Inc).

## Results

### Study Cohort Demographics and Comorbidities

**Figure 1.**
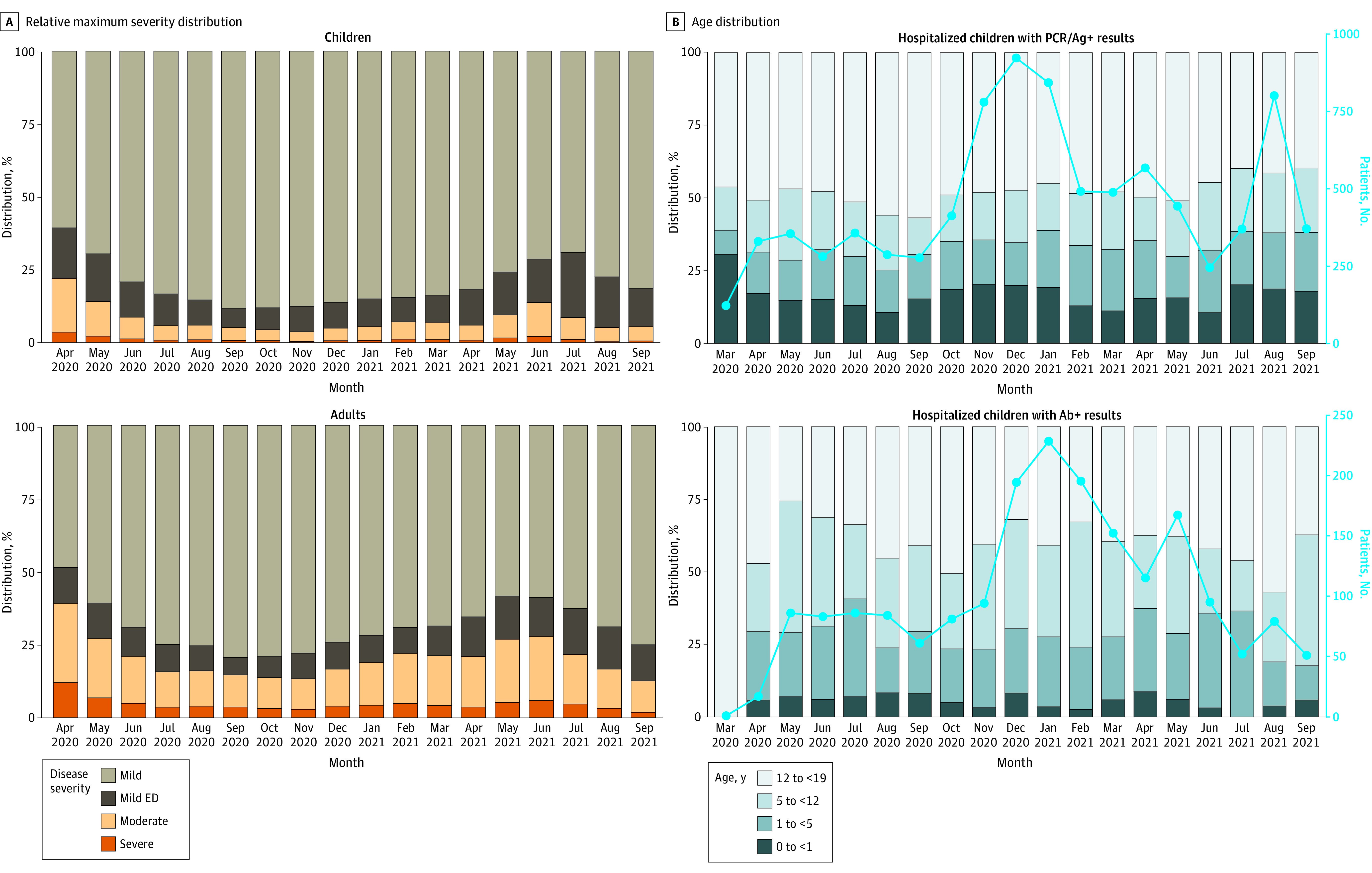
Age and Maximum Clinical Severity Distributions Over Time for Children With SARS-CoV-2 (A) Distribution of relative maximum clinical severity (by adapted World Health Organization Clinical Progression Scale categories) by month during the study period compared with adults in the National COVID Cohort Collaborative database. Severe indicates hospital mortality, invasive ventilation, vasoactive-inotropic support, or extracorporeal membrane oxygenation; moderate, hospitalized without any of the severe factors; mild ED, emergency department visit; and mild, outpatient visit. March 2020 data not shown given because there were less than 20 pediatric patients in the severe subgroup. (B) Age category distribution of children with SARS-CoV-2 infection by month during the study period stratified by test type (polymerase chain reaction [PCR]/antigen [Ag]-positive with negative or no antibody [Ab] testing vs Ab-positive regardless of PCR/Ag testing results). The light blue trendline represents monthly positive test incidence.

The N3C data set released September 24, 2021, contains 1 068 410 pediatric patients; of these, 167 262 children (15.6%) had a positive SARS-CoV-2 test result. A total of 82 882 patients (49.6%) were girls, and 83 789 were boys (49.6%); information on the biologic sex category of the remaining 591 children (0.35%) was not available (Table; eTable 1 in the Supplement). The 56 hospital systems from which these data were obtained were geographically diverse (eFigure 1 in the Supplement; eFigure 2 in the Supplement shows changes in subregion caseload over time). The incidence of positive SARS-CoV-2 pediatric encounters peaked in November 2020, with peak hospitalization incidence in December 2020 (Figure 1B).

**Table.** SARS-CoV-2 Laboratory-Confirmed Positive Pediatric Cohort Characteristics Stratified by Maximum Clinical Severity^a^^,^^b^

Variable	All pediatric encounters (N = 167 262)	Mild (n = 138 480)	Mild ED (n = 18 537)	Moderate (n = 8822)	Severe (n = 1423)	OR (95% CI)^c^	*P* value
Sex^d^							
Male	83 789 (50.1)	69 451 (50.2)	9229 (49.8)	4318 (48.9)	791 (55.6)	1.37 (1.21-1.56)^e^	<.001
Female	82 882 (49.6)	68 473 (49.4)	9290 (50.1)	4488 (50.9)	631 (44.3)		
Other	591 (0.4)	556 (0.4)	<20	<20	<20		
Age, median (IQR), y^d^	11.9 (6.0-16.1)	12.2 (6.7-16.2)	9.5 (2.6-15.5)	10.7 (2.3-16.0)	11.1 (4.7-15.5)	0.88 (0.77-1.00)	.05
Ethnicity^d^							
Hispanic or Latino	36 468 (21.8)	28 917 (20.9)	4863 (26.2)	2343 (26.6)	345 (24.2)	0.96 (0.81-1.12)	.58
Not Hispanic or Latino	105 231 (62.9)	87 012 (62.8)	12 103 (65.3)	5325 (60.4)	791 (55.6)		
Missing/unknown	25 563 (15.3)	22 551 (16.3)	1571 (8.5)	1154 (13.1)	287 (20.2)		
Race^f^						1.23 (1.04-1.45)^e^	<.01
Asian	3608 (2.2)	3037 (2.2)	356 (1.9)	179 (2.0)	36 (2.5)	NA	NA
Black or African American^g^	27 030 (16.2)	17 500 (12.6)	6823 (36.8)	2293 (26.0)	414 (29.1)	1.25 (1.06-1.47)^e^	.008
Native Hawaiian/Pacific Islander	536 (0.2)	415 (0.2)	66 (0.2)	46 (0.3)	<20 (0)	NA	NA
White	92 847 (55.5)	81 637 (59.0)	6877 (37.1)	3799 (43.1)	534 (37.5)	NA	NA
Other	39 143 (23.4)	32 640 (23.6)	3891 (21.0)	2273 (25.8)	339 (23.8)	NA	NA
Missing/unknown	4098 (2.5)	3251 (2.3)	524 (2.8)	232 (2.6)	91 (6.4)	NA	NA
Comorbidities							
Known BMI^h^	69 879 (41.8)	58 743 (42.4)	6015 (32.4)	4155 (47.1)	966 (67.9)	NA	NA
Obese (≥95th percentile)^i^	17 814 (25.5)	14 259 (24.3)	1889 (31.4)	1352 (32.5)	314 (32.5)	1.19 (1.01-1.41)^e^	.04
Asthma diagnosis	13 289 (7.9)	10 712 (7.7)	1578 (8.5)	819 (9.3)	180 (12.6)	0.91 (0.73-1.13)	.38
Diabetes diagnosis	1071 (0.6)	658 (0.5)	128 (0.7)	233 (2.6)	52 (3.7)	NA	
PCCC^j^							
Any category	23 786 (14.2)	18 386 (13.3)	2763 (14.9)	2106 (23.9)	531 (37.3)	1.20 (1.16-1.24)^e^	<.001
Congenital/genetic	6676 (4.0)	5226 (3.8)	674 (3.6)	584 (6.6)	192 (13.5)	1.17 (0.91-1.49)	.22
Cardiovascular	4800 (2.9)	3315 (2.4)	528 (2.8)	682 (7.7)	275 (19.3)	1.76 (1.40-2.22)^e^	<.001
Gastrointestinal	3538 (2.1)	2409 (1.7)	361 (1.9)	553 (6.3)	215 (15.1)	1.03 (0.75-1.43)	.85
Heme/immune	5895 (3.5)	4228 (3.1)	785 (4.2)	694 (7.9)	188 (13.2)	0.83 (0.65-1.07)	.16
Cancer	1722 (1.0)	1180 (0.9)	150 (0.8)	290 (3.3)	102 (7.2)	1.82 (1.33-2.49)^e^	<.001
Metabolic	5513 (3.3)	4098 (3.0)	587 (3.2)	628 (7.1)	200 (14.1)	1.16 (0.91-1.49)	.23
Neonatal	1850 (1.1)	1180 (0.9)	372 (2.0)	223 (2.5)	75 (5.3)	0.99 (0.70-1.41)	.95
Neuromuscular	4249 (2.5)	2924 (2.1)	473 (2.6)	614 (7.0)	238 (16.7)	1.36 (1.06-1.74)^e^	.002
Kidney	2788 (1.7)	1964 (1.4)	290 (1.6)	417 (4.7)	117 (8.2)	0.62 (0.45-0.86)^e^	.004
Respiratory	2558 (1.5)	1755 (1.3)	304 (1.6)	344 (3.9)	155 (10.9)	1.51 (1.10-2.08)^e^	.01
Technology dependence	2188 (1.3)	1194 (0.9)	252 (1.4)	505 (5.7)	237 (16.7)	1.68 (1.19-2.38)^e^	.004
Transplant	254 (0.2)	129 (0.1)	<20	71 (0.8)	37 (2.6)	1.37 (0.82-2.27)	.23

Abbreviations: BMI, body mass index; ED, emergency department; NA, not applicable; OR, odds ratio; PCCC, pediatric complex chronic condition.

^a^ Adapted from Clinical Progression Scale of the World Health Organization.^26^

^b^ Cells with less than 20 patients are censored; reported as less than 20.^16^

^c^ OR for severe disease vs moderate disease for a given variable.

^d^ The OR for sex is the odds of a male patient developing severe disease compared with a female patient. The OR for age is the odds of a patient younger than 12 years developing severe disease compared with a patient aged 12 years or older. The OR for ethnicity is the odds of a patient who is Hispanic developing severe disease vs a patient who is non-Hispanic (either not Hispanic or unknown).

^e^ Statistically significant.

^f^ The OR for a non-Black, non-White child developing severe disease compared with a White child.

^g^ The OR for a Black/African American child developing severe disease compared with a White child.

^h^ BMI calculated per Centers for Disease Control and Prevention guideline: obesity defined as any child aged 2 years or older with a BMI greater than or equal to the 95th percentile for age and sex.

^i^ Percentages of patients with a known BMI value who had a BMI greater than 95th percentile for age and sex.

^j^ PCCC comorbidities determined via prior R software implementation of PCCC to the N3C data enclave.^28,29^

The demographic characteristics of children with SARS-CoV-2 are presented in the Table, stratified by maximum clinical severity. Multivariable logistic regression showed that, among hospitalized children, those who were male (odds ratio [OR], 1.37; 95% CI, 1.21-1.56; *P* < .001), Black/African American (OR, 1.25; 95% CI, 1.06-1.47; *P* = .008), non-Black and non-White (OR, 1.23; 95% CI, 1.04-1.45; *P* = .01), obese (OR, 1.19; 95% CI, 1.01-1.41; *P* = .04), and members of several PCCC categories had higher odds of severe disease. After controlling for health care site, non-Black and non-White race and ethnicity status and the respiratory and neuromuscular PCCCs were not significant predictors (eTable 2 in the Supplement).

### Clinical Course and Illness Severity

Overall, 10 245 children (6.1%) were hospitalized. Of these, 1423 (13.9%) met the criteria for severe disease, 796 (7.8%) required mechanical ventilation, 868 (8.5%) required vasoactive-inotropic support, 42 (0.4%) required extracorporeal membrane oxygenation, and 131 (1.3%) died. The 1.3% mortality rate is consistent with 2 other pediatric reports (0.9% and 2%)^8,15^ and lower than the 11.6% rate among hospitalized adults in the N3C.^18^ Studies have reported a decrease in COVID-19 severity in adults as the pandemic progresses.^18,30^ We also observed a decrease in the proportion of children with moderate and severe disease during the study period (Figure 1A). eFigure 3 in the Supplement illustrates changes in proportions of severe disease qualification criteria over time.

### Age Distribution

Patient age distributions over time are illustrated in Figure 1B. Although most children were aged 12 to 17 years, more aged 1 to 5 and 5 to 12 years were hospitalized over time. Among hospitalized children with antibody-positive findings (regardless of PCR/Ag testing), the peak incidence occurred in January 2021 and more hospitalizations were in children aged 1 to 5 or 5 to 12 years (Figure 1B). Because antibody testing may be a surrogate for MIS-C evaluation, the timing of this peak is consistent with prior studies demonstrating maximal MIS-C risk in the 2 to 5 weeks after SARS-CoV-2 infection.^13^ Overall severity distributions for each age group are illustrated in eFigure 4 in the Supplement.

### Treatments

Antimicrobial and immunomodulatory medication use changed over time (eFigure 5 in the Supplement). As with adults in the N3C database,^18^ a higher proportion of children with severe vs moderate status received antimicrobials (987 of 1423 [69.4%] vs 2679 of 8822 [30.4%]; *P* < .001), with antibacterials (955 of 1423 [67.1%] vs 2569 of 8822 [29.1%]) and antivirals (188 of 1423 [13.2%] vs 234 of 8822 [2.7%]) being more common (*P* < .001 for all) (eTable 3 in the Supplement). Remdesivir was given to more children with severe than moderate infection (118 of 1423 [8.3%] vs 147 of 8822 [1.7%]; *P* < .001). We observed decreases in systemic antibacterial use over time, potentially related to accumulating evidence regarding the low incidence of concomitant bacterial infections.^31^

Immunomodulatory medications were more frequently given to children with severe vs moderate infection (753 of 1423 [52.9%] vs 1230 of 8822 [13.9%]), with systemic corticosteroid (684 of 1423 [48.1%] vs 1142 of 8822 [12.9%]), anakinra (153 of 1423 [10.8%] vs 89 of 8822 [1.0%]), and infliximab (39 of 1423 [2.7%] vs 52 of 8822 [0.6%]) use also more common in the severe vs moderate subgroup (*P* < .001 for all).

The US Food and Drug Administration emergency use authorization for remdesivir^32^ occurred on May 1, 2020, and the trial indicating adult survival benefit from dexamethasone^33^ and a landmark publication describing MIS-C^5^ were both published in July 2020.

### Vital Sign and Laboratory Measurements

**Figure 2.**
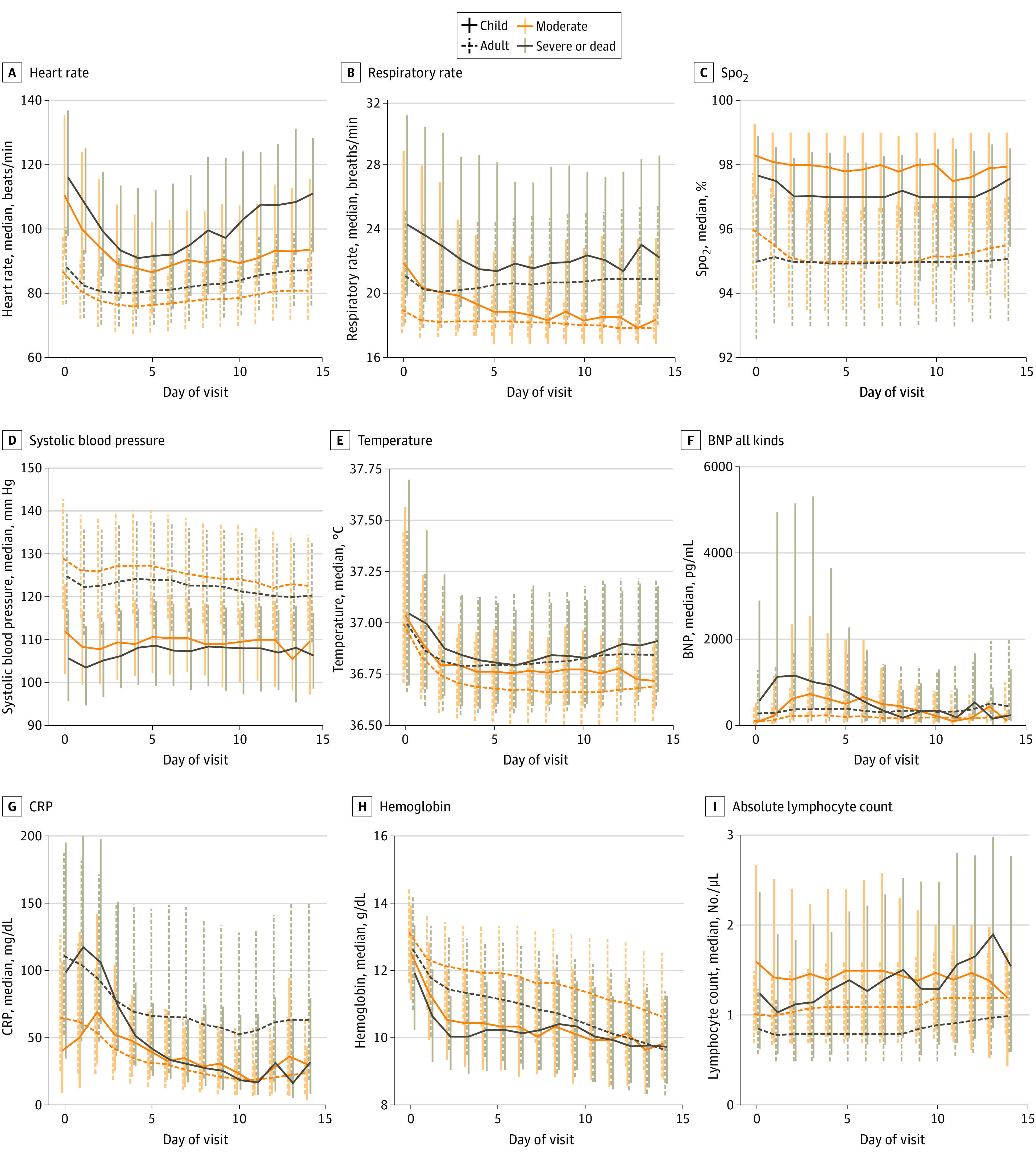
In-Hospital Vital Sign and Laboratory Value Trajectories Trajectories of selected vital sign (A-E) and laboratory (F-I) median values by day of hospitalization during pediatric hospital encounters compared with National COVID Cohort Collaborative adult values. For each day of hospitalization, the median (IQR) for patients with a given vital sign or laboratory value available on that day were calculated. The vertical bars represent the IQR for measurements of that specific vital sign or laboratory value on that day of hospitalization. SpO_2_ indicates oxygen saturation as measured by pulse oximetry. To convert brain-type natriuretic peptide (BNP) to nanograms per liter, multiply by 1; C-reactive protein to milligrams per liter, multiply by 10; hemoglobin to grams per liter, multiply by 10; and lymphocytes to ×10^9^ per liter, multiply by 0.001.

Compared with the moderate severity subgroup, the severe subgroup had more abnormal initial (day 0) values for many vital signs, including systolic and diastolic blood pressure (lower), oxygen saturation as measured by pulse oximetry (lower), heart rate (higher), and respiratory rate (higher) (eTable 4 in the Supplement). We saw statistically significant improvements (values becoming more within the reference ranges) in most vital signs when comparing day 0 with day 7 for both severity subgroups (Figure 2).

Children who eventually experienced the highest maximum clinical severity also had many laboratory test results with initial values that were more abnormal than in the moderate severity subgroup (eTable 5 in the Supplement). Specifically, initial median values were more abnormal in the severe subgroup for several tests demonstrative of organ dysfunction (alanine aminotransferase and aspartate aminotransferase [higher], brain-type natriuretic peptide [higher], creatinine [higher], and platelets [lower]), and inflammation (albumin [lower], d-dimer [higher], ferritin [higher], C-reactive protein [higher], and procalcitonin [higher]) (eTable 5 in the Supplement). Compared with hospital day 0, laboratory values on day 7 changed in a statistically significant way with values increasingly within the reference ranges for most tests within both severity subgroups (Figure 2; eTable 5 in the Supplement). eTable 6 in the Supplement presents the proportions of patients with each laboratory result available. The improving trajectories for most vital sign and laboratory values likely reflect the low mortality rate and high recovery rate of the children. eFigure 6 in the Supplement provides additional vital sign and laboratory trajectory results.

### Acute COVID-19 vs MIS-C

**Figure 3.**
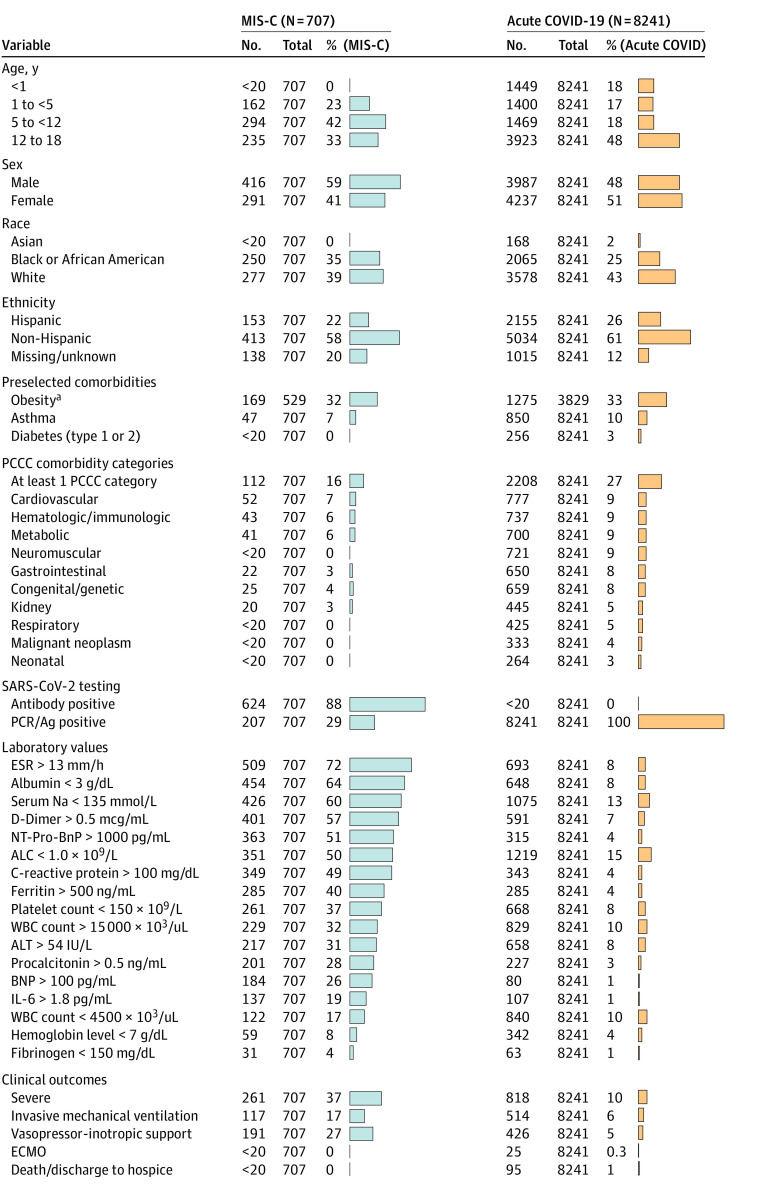
Characteristics and Outcomes of Children Hospitalized With Multisystem Inflammatory Syndrome in Children (MIS-C) vs Acute COVID-19 Comparison of the percentage of hospitalized children with MIS-C (identified via the presence of qualifying *International Statistical Classification of Diseases, 10th Revision* code) and acute COVID-19 (positive SARS-CoV-2 PCR/Ag but no positive antibody test or MIS-C *International Statistical Classification of Diseases, 10th Revision* code) with a given demographic characteristic, preexisting comorbidity, abnormal laboratory test value (during hospitalization), or clinical outcome. eTable 7 in the Supplement provides the absolute number of patients in each corresponding category. Per National COVID Cohort Collaborative policy, groups with fewer than 20 patient encounters were censored and are reported as 0%. ALC indicates absolute lymphocyte count; ALT, alanine aminotransferase; ANC, absolute neutrophil count; BNP, brain-type natriuretic peptide; ECMO, extracorporeal membrane oxygenation; ESR, erythrocyte sedimentation rate; IL-6, interleukin 6; Na, sodium; NT-Pro-BnP, N-terminal pro b-type natriuretic peptide; PCCC, pediatric complex chronic condition; PCR/Ag, polymerase chain reaction/antigen; and WBC, white blood cell. To convert ALC to ×10^9^ per liter, multiply by 0.001; ALT to microkatals per liter, multiply by 0.0167; ANC to ×10^9^ per liter, multiply by 0.001; BNP to nanograms per liter, multiply by 1; ferritin to micrograms per liter, multiply by 1; and Na to millimoles per liter, multiply by 1. ^a^The percentage of children with obesity was calculated by dividing the number of children aged 2 years or older with a body mass index (for age and sex) greater than or equal to the 95th percentile by the number of children in that subgroup who were aged 2 years or older and had a body mass index measurement available.

We identified 707 children with MIS-C of whom 261 (36.9%) met the criteria for severe disease. Demographic characteristics, laboratory results, and clinical outcomes for children with MIS-C vs acute COVID-19 are shown in Figure 3. A multivariable logistic regression model demonstrated that male sex (OR, 1.59; 95% CI, 1.33-1.90; *P* < .001), age younger than 12 years (OR, 1.81; 95% CI, 1.51-2.18; *P* < .001), Black/African American race (OR, 1.44; 95% CI, 1.17-1.77; *P* < .001), obesity (OR, 1.76; 95% CI, 1.40-2.22; *P* < .001), and not having a PCCC (OR, 0.72; 95% CI, 0.65-0.80; *P* < .001) were associated with increased odds of MIS-C diagnosis vs acute COVID-19. Compared with children with acute COVID-19 infection, a higher proportion of children with MIS-C required vasoactive-inotropic support (191 of 707 [27.0%] vs 426 of 8241 [5.2%]; *P* < .001) and invasive ventilation (117 of 707 [16.5%] vs 514 of 8241 [6.2%]; *P* < .001) (eTable 7 in the Supplement). In addition, for all 18 preselected laboratory tests, the MIS-C group had a higher proportion of children with abnormal values compared with the acute COVID-19 group.

### Delta Era vs Pre-Delta Era Comparisons

**Figure 4.**
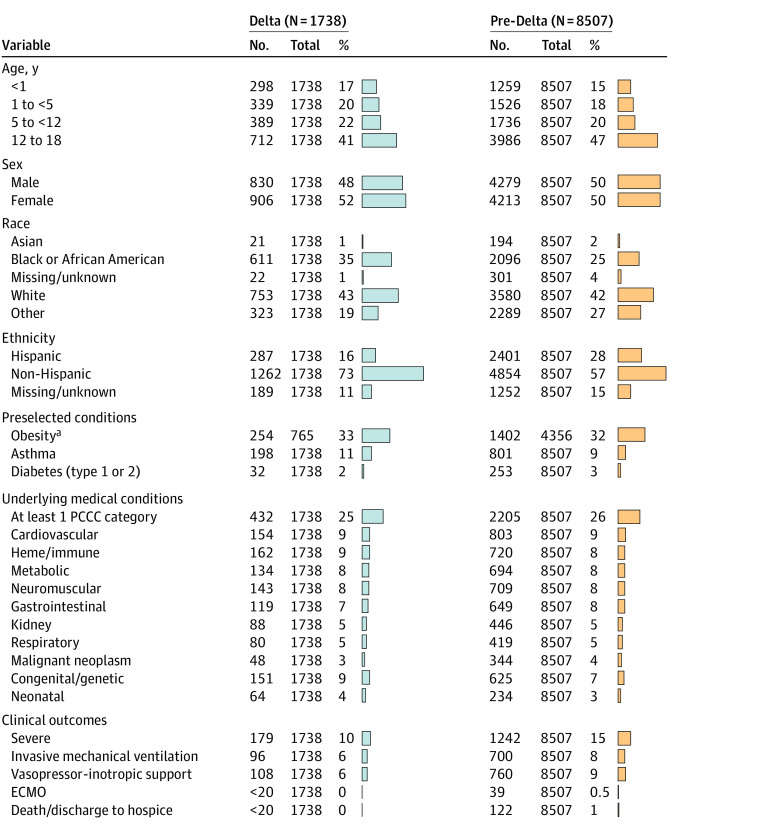
Characteristics and Outcomes of Children in the Pre-Delta and Delta Eras Comparison of the percent of children in the pre-Delta era (before June 26, 2021) to the Delta era with a given demographic characteristic, comorbidity, abnormal laboratory value during hospitalization, or clinical outcome. eTable 8 in the Supplement reports the absolute number of patients in each corresponding category. Per National COVID Cohort Collaborative policy, groups with less than 20 patient encounters were censored and are reported as 0%. ^a^The percentage of children with obesity was calculated by dividing the number of children aged 2 years or older with a body mass index (for age and sex) greater than or equal to the 95th percentile by the number of children in that subgroup who were aged 2 years or older and had a body mass index measurement available.

Of the 29 191 Delta era encounters, 1738 children (6.0%) were hospitalized compared with 8507 of 138 071 children (6.2%) in the pre-Delta era (*P* = .18). There were 1242 children (14.6%) in the pre-Delta era and 179 children (10.3%) in the Delta era with severe disease. A multivariable logistic regression model demonstrated the odds of severe vs moderate disease decreased by a factor of 0.67 (95% CI, 0.57-0.79; *P* < .001) during the Delta era. Multivariable logistic regression demonstrated an increase in the odds of severe disease (ΔOR, 1.69; 95% CI, 1.02-2.82; *P* = .04) for children classified as non-Black, non-White during the Delta era (Figure 4; eTable 8 in the Supplement).

## Discussion

Using the largest US pediatric SARS-CoV-2 cohort at the time of the study, we addressed a gap in the pediatric SARS-CoV-2 literature: a description of vital sign and laboratory values and trajectories during hospitalization among children with SARS-CoV-2 infection with varying peak clinical severity. Although reports from Feldstein et al^5,6^ include median laboratory results from children with MIS-C and acute COVID-19, to our knowledge, ours is the first report describing vital sign and laboratory trajectories during pediatric SARS-CoV-2 hospitalization with specific attention to maximum clinical severity. We observed differences in initial vital sign and laboratory values between severity subgroups. Many differences were subtle and without obvious clinical implication. When combined with the identified demographic characteristic and comorbidity risk factors, these results suggest that early identification of children likely to progress to a more severe phenotype could be achieved using readily available data from the day of admission. Future work may include design of predictive models and clinical decision support tools able to use these often subtle differences to aid in early identification of children at risk for subsequent deterioration.

We also report on hospitalization trends, age and severity distributions, and treatment regimens throughout the study period. The 6.1% hospitalization rate in our cohort is similar to the 7% rate reported by Bailey et al,^15^ although lower than reported by Kompaniyets et al^8^ (9.9%) and Preston et al^3^ (12%). Our cohort’s median age (11.9 years) was similar in the Kompaniyets et al^8^ study (12 years) as was the prevalence of comorbid asthma (7.9% vs 10.2%) and neurologic/neuromuscular disease (2.5% vs 3.9%). Our cohort’s rate of mechanical ventilation (7.8%) among hospitalized children was similar to 2 prior reports (7% and 6%),^7,8^ although less than in Belay et al^13^ (14.6% in the COVID-19 cohort). Similar to Götzinger et al^7^ and Preston et al,^3^ we observed that male sex and chronic medical conditions (PCCCs in this study) were risk factors for severe disease. We found that Black/African American race and obesity were also associated with higher maximum clinical severity once the children were hospitalized.

Although Shekerdemian et al^10^ and Feldstein et al^6^ both reported use of various antimicrobial and immunomodulatory medications in pediatric SARS-CoV-2, to our knowledge, ours is the first report describing changes in medication use over time. In this study, systemic antimicrobials and immunomodulatory medications were frequently used in the severe subgroup. The high frequency of corticosteroid use, specifically, is likely associated with emerging evidence of efficacy for treatment of severe COVID-19 and MIS-C.^33,34^

As others have reported,^6,13,35^ compared with children with acute COVID-19, we found that being male, Black/African American, younger than 12 years, and not having preexisting comorbidities (a PCCC) were associated with increased odds of MIS-C diagnosis. We also found obesity to be a significant MIS-C predictor. Children with MIS-C demonstrated a severe clinical phenotype with significant laboratory evidence of inflammation and frequent need for invasive ventilation and vasopressor-inotropic support.

Given the relatively recent introduction of *International Statistical Classification of Diseases, 10th Revision* codes for MIS-C (and unknown consistency of use), sensitivity of the codes for MIS-C is uncertain. However, in the setting of hospitalization with a positive SARS-CoV-2 test, the positive predictive value is likely to be high. The MIS-C incidence we describe is likely a conservative estimate. A recent study by Geva et al^35^ used unsupervised clustering techniques to distinguish MIS-C from acute COVID-19 and identified many clinical and laboratory indicators similar to those highlighted in this study. Additional work is needed to develop and validate computable phenotypes for identification of MIS-C cases for subsequent research.

Clinical outcomes (eg, rates of mechanical ventilation) and medication use in acute COVID-19 and MIS-C have varied between studies.^6,7,8,10,15,36^ Many reports of pediatric acute COVID-19 and MIS-C originate from single centers or health systems.^2,4,36,37,38,39^ Recently, several large-scale studies reported risk factors and outcomes for pediatric COVID-19^3,7,8,10,15^ and MIS-C.^6,13^ However, none have included analysis of highly granular, patient-level data to compare trajectories of individual vital sign and laboratory values between different clinical severity subgroups. Such analyses improve the capacity to build SARS-CoV-2–specific predictive models and clinical decision support tools and allowed for successful testing of our 5 original hypotheses.

In addition, this report provides preliminary data on differences among children infected with SARS-CoV-2 in the era prior to Delta variant dominance compared with those in the Delta era. We found a similar hospitalization rate and that odds for severe vs moderate disease decreased during the Delta era. This decrease may reflect that more previously healthy children were hospitalized in the Delta era.

### Limitations

This study has limitations. Because our data were aggregated from many health systems using 4 different common data models that vary in granularity, some sites may have systematic missingness of certain variables. In addition, some respiratory data (oxygen flow, fraction of inspired oxygen, and specific ventilator settings) are not fully available. Because the exact timing of laboratory specimens is inconsistently provided by sites, laboratory findings were standardized to a calendar day.

With high rates of asymptomatic to minimally symptomatic pediatric infections and increasing adoption of universal SARS-CoV-2 testing policies for pediatric hospital admissions, we cannot definitively attribute reasons for hospital admissions (SARS-CoV-2 vs another unrelated cause). Because many hospitalized children were not given antimicrobials or immunomodulatory medications, admission may have been due to non–SARS-CoV-2 conditions. Inability to definitively link SARS-CoV-2 infection to the need for hospitalization may limit interpretation of variables associated with higher clinical severity because care for pre-existing comorbidities in children with medically complex conditions could be difficult to distinguish from management of SARS-CoV-2 sequelae. We also were not able to account for the differing reference ranges for vital signs and many laboratory findings known to vary throughout childhood. In addition, the diagnostic organ dysfunction criteria for MIS-C require, by definition, a high-acuity phenotype, and MIS-C clinical outcomes should be interpreted with this in mind.

## Conclusions

This cohort study reports the characteristics and outcomes of what is, to our knowledge, the largest US cohort of children with SARS-CoV-2 to date. The N3C database provides a diverse, granular view of pediatric SARS-CoV-2 infections and allows for novel vital sign and laboratory value trajectory mapping. Further work is warranted to optimize translation of this knowledge into improved clinical care.

## Notes

Supplement. **eMethods.** Detailed Methods **eTable 1.** N3C Pediatric and Adult Cohort Characteristics **eTable 2.** Adjusted Odds for Severe Disease by Demographic or Comorbidity Variable **eTable 3.** Medication Administration Rates by Pediatric Severity Subgroup **eTable 4.** Vital Sign Changes During the First Week of Hospitalization **eTable 5.** Changes in Laboratory Test Values During the First Week of Hospitalization **eTable 6.** Proportion of Children With a Given Lab Test Result by Severity Subgroup **eTable 7.** MIS-C Versus Acute COVID-19 Characteristics, Outcomes, and Risk Factors **eTable 8.** Delta Versus Pre-Delta Differences in Patient Characteristics, Outcomes, and Severity Risk Factors **eFigure 1.** Geographic Distribution and Case Incidence Over Time N3C Pediatric Patients **eFigure 2.** Subregion Positive Test Result Trends by Test Type **eFigure 3.** Change in Qualifying Criteria for “Severe” Subgroup Classification Over Time **eFigure 4.** Maximum Clinical Severity Distributions Among Prespecified Age Groups **eFigure 5.** Antimicrobial and Immunomodulatory Medication Usage Trends **eFigure 6.** Additional Vital Sign and Laboratory Value In-Hospital Trajectories
eReferences
