# Supplementary material for: Characteristics, Outcomes, and Severity Risk Factors Associated With SARS-CoV-2 Infection Among Children in the US National COVID Cohort Collaborative
Source: JAMA Netw Open. 2022 Feb 8;5(2):e2143151. doi: 10.1001/jamanetworkopen.2021.43151 (PMC8826172; doi:10.1001/jamanetworkopen.2021.43151)
Supplement: Supplement. — eMethods. Detailed Methods eTable 1. N3C Pediatric and Adult Cohort Characteristics eTable 2. Adjusted Odds for Severe Disease by Demographic or Comorbidity Variable eTable 3. Medication Administration Rates by Pediatric Severity Subgroup eTable 4. Vital Sign Changes During the First Week of Hospitalization eTable 5. Changes in Laboratory Test Values During the First Week of Hospitalization eTable 6. Proportion of Children With a Given Lab Test Result by Severity Subgroup eTable 7. MIS-C Versus Acute COVID-19 Characteristics, Outcomes, and Risk Factors eTable 8. Delta Versus Pre-Delta Differences in Patient Characteristics, Outcomes, and Severity Risk Factors eFigure 1. Geographic Distribution and Case Incidence Over Time N3C Pediatric Patients eFigure 2. Subregion Positive Test Result Trends by Test Type eFigure 3. Change in Qualifying Criteria for “Severe” Subgroup Classification Over Time eFigure 4. Maximum Clinical Severity Distributions Among Prespecified Age Groups eFigure 5. Antimicrobial and Immunomodulatory Medication Usage Trends eFigure 6. Additional Vital Sign and Laboratory Value In-Hospital Trajectories eReferences [file jamanetwopen-e2143151-s001.pdf]

Supplementary Online Content

Martin B, DeWitt PE, Russell S, et al. Characteristics, outcomes, and severity risk factors associated with SARS-CoV-2 infection among children in the US National COVID Cohort Collaborative. *JAMA Netw Open*. 2022;5(2):e2143151. doi:10.1001/jamanetworkopen.2021.43151

eMethods. Detailed Methods

eTable 1. N3C Pediatric and Adult Cohort Characteristics

eTable 2. Adjusted Odds for Severe Disease by Demographic or Comorbidity Variable

eTable 3. Medication Administration Rates by Pediatric Severity Subgroup

eTable 4. Vital Sign Changes During the First Week of Hospitalization

eTable 5. Changes in Laboratory Test Values During the First Week of Hospitalization

eTable 6. Proportion of Children With a Given Lab Test Result by Severity Subgroup

eTable 7. MIS-C Versus Acute COVID-19 Characteristics, Outcomes, and Risk Factors

eTable 8. Delta Versus Pre-Delta Differences in Patient Characteristics, Outcomes, and Severity Risk Factors

eFigure 1. Geographic Distribution and Case Incidence Over Time N3C Pediatric Patients

eFigure 2. Subregion Positive Test Result Trends by Test Type

eFigure 3. Change in Qualifying Criteria for “Severe” Subgroup Classification Over Time

eFigure 4. Maximum Clinical Severity Distributions Among Prespecified Age Groups

eFigure 5. Antimicrobial and Immunomodulatory Medication Usage Trends

eFigure 6. Additional Vital Sign and Laboratory Value In-Hospital Trajectories

eReferences

This supplementary material has been provided by the authors to give readers additional information about their work.

© 2022 Martin B et al. *JAMA Network Open.*

eMethods. Detailed Methods

N3C Structure

As described in prior work[1-3], the N3C is housed in a cloud-based, secure, data enclave managed by the National Center for Advancing Translational Sciences (NCATS) to which contributing partner sites submit a limited data set in one of four common data models (CDMs). Thereafter, sites continue to provide weekly data updates. N3C harmonizes all data into the Observational Medical Outcomes Partnership (OMOP) CDM[4]. See Haendel et al.[3] for details regarding data transfer, harmonization, and data quality assurance and integration. Computable phenotypes for COVID-19 and N3C COVID-negative controls are publicly available on GitHub[1].

N3C Patients and Prior Medical History Data

N3C includes cases, controls, and additional non-SARS-CoV-2 related patient encounters (both inpatient and outpatient) all available for analysis. All N3C cases and demographically matched (by age, sex, race, and ethnicity) SARS-CoV-2 negative controls are drawn from encounters in the database starting on or after 1/1/2020. Positive cases are defined via a priori defined SARS-CoV-2 testing and diagnosis code criteria. Specifically, an N3C SARS-CoV-2 case includes any patient from an N3C study site with an encounter on or after 1/1/2020 meeting the following requirements:

1) one of a set of a priori-defined SARS-CoV-2 laboratory tests,
2) a "strong positive" diagnostic code, or
3) two "weak positive" diagnostic codes during one encounter or on the same date prior to 5/1/2020.

This case definition is publicly available on GitHub[1]. Encounters in the same health system on or after 1/1/2018 are also included to provide relevant clinical information on pre-existing health conditions ("lookback data"). For the purposes of our analysis

Controls within the database (not used for the purposes of this work's analysis) include patient encounters with a negative SARS-CoV-2 test (and no positive SARS-CoV-2 testing) who are matched by age, sex, race, and ethnicity to the SARS-CoV-2 positive cases. Data for all other patient encounters from the N3C sites during the study period (including many children without any SARS-CoV-2 testing) during the study period are also available within the N3C database for analysis.

Index Encounter and Clinical Severity Definition

The N3C index encounter assigned to a child with a positive SARS-CoV-2 test result was identified via evaluation of all encounters starting up to 30 days before through 7 days after each positive test. We selected as the index encounter that which, amongst this encounter set, demonstrated the maximum Clinical Progression Scale (CPS) score (created by the World Health Organization [WHO] for COVID-19 clinical research[5]). Of note, we combined certain WHO CPS categories due to data limitations (e.g., certain sites did not submit fraction of inspired oxygen [FiO_2]), resulting in four separate outcome groups:

1) Mild (outpatient visit only)
2) Mild ED (outpatient with emergency department visit)
3) Moderate (hospitalized without requiring invasive ventilation, vasopressors/inotropes, or ECMO)
4) Severe (hospitalized and requiring invasive ventilation, vasopressors/inotropes, or ECMO, or death)

As such, we identified a single encounter for each SARS-CoV-2 positive pediatric patient for cohort analysis. All subsequent visit-level analyses were performed using this select encounter only.

Comorbidity Identification

All concept sets developed for and utilized within this report are freely available on the platform and include input from clinical subject matter and informatics experts. All concept sets and analytic pipelines are fully reproducible and will be made publicly available. Children with asthma, obesity, and diabetes mellitus as well as those who received vasoactive-inotropic support were identified via adaptation and application of existing concept sets within the enclave. Children with a pediatric complex chronic condition[6] (PCCC) were identified using an adapted version of our prior PCCC R implementation[7].

© 2022 Martin B et al. *JAMA Network Open.*

Additional Software Packages Used
We used a variety of SQL, python, and R packages to clean and analyze data. We utilized SparkR[8] and pyspark[9] to interface with Apache Spark[10] and utilized R's ggplot2[11] for all visualizations.

Additional Study Methods (as per the STROBE[12] guidelines):
Study Design: Prospective cohort study of pediatric patients within the N3C

Setting: Of the 90 healthcare sites that signed data transfer agreements (DTAs) with the N3C, 65 contributed data to the N3C enclave. Among these, 56 sites contributed data which 1) completed harmonization and integration, 2) was released for analysis, and 3) included the necessary death and mechanical ventilation information. Each of these 56 data partners represents an individual healthcare entity (e.g. clinic, hospital, healthcare center, or healthcare network). Many of these include large health systems with numerous, separate primary care, urgent care, and inpatient care locations. All subsequent patient encounters at each site beginning after the N3C join date are included along with relevant lookback data for each patient among the contributed data (includes encounters from each participating site starting on or after 1/1/2018).

Participants: All children <19-years-old at the time of their first SARS-CoV-2 test within the N3C with an encounter end date prior to 9/24/2021.

Variables:
Patient Measurements:
- Date/time of viral testing
- Geographic location at the time of viral testing by United States Census Bureau geographic subregion
- Demographics: EHR-documented race, ethnicity, biological sex, and age (at encounter start)
- Preselected comorbidities: asthma*, diabetes mellitus*, obesity†, and pediatric complex chronic condition (PCCC, as defined by Chris Feudtner et al[6] and determined via adaptation of our R implementation[7] using available diagnosis and procedure codes)
- Medication administration for each patient with matching day of hospitalization (day 0 = day of admission)
- Select patient vital sign and laboratory values documented during index encounter hospitalization

*Asthma and diabetes mellitus diagnoses were identified via application of validated, pre-existing concept sets within the N3C enclave.
†Presence of obesity is determined only for those children ≥2-years-old with a BMI measurement available, and obesity is defined as those with a BMI ≥95th percentile for age and sex per CDC criteria[13]).

Outcomes Variables:
- Severe versus moderate maximum CPS clinical severity category
- MIS-C versus acute COVID-19 patient class
- Severe versus moderate maximum CPS clinical severity category by variant era (Delta era versus pre-Delta era)
    - Delta era is defined as all encounters with a start date on or after 6/26/2021, the approximate date at which Delta variant strains exceeded 50% of all sampled strains over a two week period[14]

Data Sources / Measurement:
56 data partners from the 9/24/2021 release of the N3C dataset. See Haendel et al.[3] for details on data ingestion, common data model mapping, quality assurance, and harmonization.

Bias:
To assess for the introduction of bias by contributing site, we performed a sensitivity analysis to evaluate the impact of the data source (that is, the contributing healthcare site) on the strength of association between each variable and patient maximum clinical severity. For each of the above listed outcome variables, a sensitivity analysis was performed to identify changes in the strength of association between a given predictor and the outcome of interest (see "Statistical Methods" section below).

Study Size:
The study size was determined by the number of index patient encounters (see above) identified at each contributing data partner during the study period.

Quantitative Variables:
Quantitative variables such as patient age and BMI are reported as medians with associated interquartile range (IQR). Patients are grouped based on index encounter maximum clinical severity (e.g. mild, mild ED, moderate, or severe) to aid in identification of variables associated with higher peak clinical severity. For vital sign measurements obtained during hospitalization, each vital sign is reported using the mean for that day (e.g. day 0) over all patients in a given maximum clinical severity category with its corresponding 95% confidence interval. Similarly, laboratory test values are reported using the mean for each lab on a given day of hospitalization over all patients with the corresponding 95% confidence interval and grouped by maximum clinical severity category. When vital signs and laboratory test values are plotted by day of hospitalization, the median is shown along with the associated IQR for that day.

Statistical Methods
As noted above, quantitative variables are reported as medians with corresponding interquartile ranges. Categorical variables (e.g. ethnicity, race, or presence of a particular PCCC category) are reported as the number of patients and associated percentage, stratified by maximum clinical severity category. We used chi square deviance tests to evaluate for statistically significant changes in patient severity over time (specifically the odds of moderate or severe disease) and statistically significant change in the relative proportions of preselected age groups over time. Proportions of children in each maximum clinical severity category receiving a given medication or medication class (e.g. corticosteroid) were compared using Pearson’s chi-squared test statistic. To report on the percent of hospitalized patients receiving a given medication or medication class, patients were grouped by study quarter (three month blocks), by index encounter start date, to allow for some granularity while ensuring that each datapoint represented a patient group of at least 20 patients (as reporting of results for groups comprised of <20 patients is not allowed per N3C policy). Separate lines for pediatric and adult classes were plotted to highlight changes in medication use over time during the study period within these two groups.

Multivariable logistic regression (MLR) was used to identify which predictors among a set of preselected variables were associated with an increased odds of severe (versus moderate) maximum clinical severity. Preselected variables included biologic sex, age, ethnicity, race, asthma, obesity, and PCCC categories. Diabetes mellitus (both type I and type II) was initially selected as a predictor for evaluation; however, the low number of hospitalized children with diabetes precluded evaluation in an MLR model. To evaluate age as a risk factor, outcomes of children under 12-years-old were compared to those of children 12-years-old and over. To evaluate ethnicity as a risk factor for severe disease, Hispanic / Latino patient outcomes were compared with non-Hispanic / non-Latino patient outcomes. Similarly, to evaluate race as a risk factor for severe disease, Black / African American and non-Black, non-White severity outcomes were each compared to those of White children. To evaluate the impact of obesity, an MLR for severe (versus moderate) disease was created using only those children ≥2-years-old with a BMI measurement available.

This same MLR approach was used to identify predictors associated with increased odds of receiving an MIS-C diagnosis (versus acute COVID-19) and to identify changes in odds of severe (versus moderate) disease in the Delta era as compared to the pre-Delta era (both overall and for individual predictors). Pre-Delta era and Delta era odds ratios (and 95% CIs) for severe disease for each predictor were calculated along with the change in odds (and corresponding 95% CI) for the change. For each MLR, a separate sensitivity analysis was performed in which we used generalized estimating equations (GEEs) to account for the impact of patient healthcare site on the odds ratio for severe disease for each predictor. All GEEs used an exchangeable working correlation structure.

Mean values for a given vital sign or laboratory test value on a given day of hospitalization (e.g. day 0 or day 7) were compared using GEEs with an exchangeable working correlation structure. We assessed for significance in change over time for each vital sign or lab result during hospitalization using a linear model to average over the first week of values. We then used the model results to estimate the difference between hospital day 0 and day 7 for each severity subgroup. Lastly, proportions of cases requiring hospitalization in the pre-Delta and Delta eras were compared using chi-square tests.

© 2022 Martin B et al. *JAMA Network Open.*

Data Sharing

The N3C Data Enclave (covid.cd2h.org/enclave) houses fully reproducible, transparent, and broadly available limited and de-identified datasets (HIPAA definitions available at https://www.hhs.gov/hipaa/index.html). Enclave data can be accessed by investigators at institutions that have signed a Data Use Agreement with the NIH who have taken the required human subjects and security training and agree to the N3C User Code of Conduct. Those desiring access to the limited dataset need also supply an IRB protocol from their institution. Data access requests are reviewed by the NIH Data Access Committee. A full description of the N3C Enclave governance has been published[3] and additional information on the access application process is available on the NCATS website: https://ncats.nih.gov/n3c/about/applying-for-access. The data model utilized is OMOP 5.3.1, and specifications are available at: https://ncats.nih.gov/files/OMOP_CDM_COVID.pdf

Additional N3C resources including code and governance documents are available within the project GitHub repositories and/or in Zenodo for archival purposes: https://github.com/National-COVID-Cohort-Collaborative https://zenodo.org/communities/cd2h-covid/

Additional information regarding each of the source Common Data Models is available at:
OHDSI: https://ohdsi.org/
PCORNet: https://pcornet.org/
ACT: https://www.dbmi.pitt.edu/node/53983
TriNetX: https://trinetx.com/

© 2022 Martin B et al. *JAMA Network Open.*

Ethics and Regulatory
The N3C data transfer to NCATS is performed under a Johns Hopkins University Reliance Protocol # IRB00249128 or individual site agreements with NIH. Use of the N3C data for this study is authorized under the following IRB Protocols:

**Site**	**IRB name**	**Exempted vs approved**	**Protocol number**
University of Colorado	Colorado Multiple Institutional Review Board	approved	20-2225
Johns Hopkins University	Johns Hopkins Office of Human Subjects Research - Institutional Review Board	approved	IRB00249128
University of North Carolina	University of North Carolina Chapel Hill Institutional Review Board	exempted	20-3106
Stony Brook University	Office of Research Compliance, Division of Human Subject Protections, Stony Brook University	exempted	IRB2020-00604

© 2022 Martin B et al. *JAMA Network Open.*

eTable 1: N3C Pediatric and Adult Cohort Characteristics

A. Pediatric Patients

Demographic Category	All Children Tested	PCR/Ag Positive	PCR/Ag Negative	PCR/Ag UNK	No PCR / Ag Test	Ab Positive	Ab Negative	Ab UNK	No Ab Test
Number of Encounters	1,068,410	160,166	655,422	155	252,667	7,579	9,399	<20	1,051,425
Age									
Age(y), mean (SD)	9.7 (5.9)	10.9 (5.8)	9.2 (5.9)	9.2 (5.7)	10.1 (5.9)	11.2 (5.6)	11.1 (5.7)	*	9.7 (5.9)
Age(y), median (IQR)	9.8 (4.1, 15.2)	11.9 (6.0, 16.1)	9.0 (3.7, 14.8)	8.5 (4.8, 13.8)	10.4 (4.5, 15.7)	12.3 (6.4, 16.2)	12.3 (6.2, 16.2)	*	9.7 (4.1, 15.2)
Gender									
Male	542,779 (50.8%)	80,180 (50.1%)	337,987 (51.6%)	69 (44.5%)	124,543 (49.3%)	3,873 (51.1%)	4,880 (51.9%)	<20 (0%)	534,021 (50.8%)
Female	514,972 (48.2%)	79,407 (49.6%)	314,634 (48.0%)	78 (50.3%)	120,853 (47.8%)	3,694 (48.7%)	4,484 (47.7%)	<20 (0%)	506,792 (48.2%)
Other	10,659 (1.0%)	579 (0.4%)	2,801 (0.4%)	<20 (0%)	7,271 (2.9%)	<20 (0%)	35 (0.4%)	<20 (0%)	10,612 (1.0%)
Ethnicity									
Hispanic or Latino	201,162 (18.8%)	34,899 (21.8%)	124,267 (19.0%)	45 (29.0%)	41,951 (16.6%)	1,699 (22.4%)	1,467 (15.6%)	<20 (0%)	197,993 (18.8%)
Not Hispanic or Latino	702,394 (65.7%)	100,836 (63.0%)	442,833 (67.6%)	92 (59.4%)	158,633 (62.8%)	4,671 (61.6%)	6,525 (69.4%)	<20 (0%)	691,197 (65.7%)
Missing / Unknown	164,854 (15.4%)	24,431 (15.3%)	88,322 (13.5%)	<20 (0%)	52,083 (20.6%)	1,209 (16.0%)	1,407 (15.0%)	<20 (0%)	162,235 (15.4%)
Race									
Asian	26,622 (2.5%)	3,450 (2.2%)	17,092 (2.6%)	<20 (0%)	6,077 (2.4%)	168 (2.2%)	271 (2.9%)	<20 (0%)	26,183 (2.5%)
Black or African American	172,861 (16.2%)	26,168 (16.3%)	109,130 (16.7%)	26 (16.8%)	37,537 (14.9%)	977 (12.9%)	1,250 (13.3%)	<20 (0%)	170,633 (16.2%)
Native Hawaiian / Pacific Islander	2,919 (0.3%)	380 (0.2%)	1,738 (0.3%)	<20 (0%)	801 (0.3%)	<20 (0%)	29 (0.3%)	<20 (0%)	2,874 (0.3%)
White	601,095 (56.3%)	89,696 (56.0%)	385,091 (58.8%)	79 (51.0%)	126,229 (50.0%)	3,345 (44.1%)	5,249 (55.8%)	<20 (0%)	592,498 (56.4%)
Other	237,079 (22.2%)	36,346 (22.7%)	131,173 (20.0%)	46 (29.7%)	69,514 (27.5%)	2,935 (38.7%)	2,468 (26.3%)	<20 (0%)	231,673 (22.0%)
Missing / Unknown	27,312 (2.6%)	3,990 (2.5%)	10,852 (1.7%)	<20 (0%)	12,469 (4.9%)	128 (1.7%)	117 (1.2%)	<20 (0%)	27,067 (2.6%)

© 2022 Martin B et al. *JAMA Network Open.*

B. Adult Patients									
**Demographic Category**	**All Adults Tested**	**PCR/Ag Positive**	**PCR/Ag Negative**	**PCR/Ag UNK**	**No PCR/Ag Test**	**Ab Positive**	**Ab Negative**	**Ab UNK**	**No Ab Test**
Number of Encounters	6,149,156	929,785	3,584,274	863	1,634,234	93,890	148,073	154	5,907,039
Age									
Age(y) mean (SD)	49.8 (18.5)	48.0 (18.4)	50.5 (18.5)	47.2 (17.9)	49.4 (18.3)	51.8 (16.9)	50.2 (16.7)	45.4 (13.7)	49.8 (18.5)
Age(y) median (IQR)	49.5 (34.1, 63.8)	46.7 (32.2, 61.3)	50.5 (34.6, 64.7)	45.4 (32.5, 60.4)	48.9 (34.0, 63.0)	52.3 (38.2, 64.3)	49.9 (36.5, 62.4)	46.5 (34.7, 53.5)	49.4 (33.9, 63.8)
Gender									
Male	2,630,622 (42.8%)	417,987 (45.0%)	1,529,245 (42.7%)	333 (38.6%)	683,057 (41.8%)	37,408 (39.8%)	59,210 (40.0%)	66 (42.9%)	2,533,938 (42.9%)
Female	3,422,386 (55.7%)	505,600 (54.4%)	2,026,790 (56.5%)	432 (50.1%)	889,564 (54.4%)	55,996 (59.6%)	88,209 (59.6%)	88 (57.1%)	3,278,093 (55.5%)
Other	96,148 (1.6%)	6,198 (0.7%)	28,239 (0.8%)	98 (11.4%)	61,613 (3.8%)	486 (0.5%)	654 (0.4%)	<20 (0%)	95,008 (1.6%)
Ethnicity									
Hispanic or Latino	703,632 (11.4%)	154,384 (16.6%)	388,108 (10.8%)	157 (18.2%)	160,983 (9.9%)	13,481 (14.4%)	15,209 (10.3%)	<20 (0%)	674,934 (11.4%)
Not Hispanic or Latino	4,730,166 (76.9%)	669,372 (72.0%)	2,881,057 (80.4%)	557 (64.5%)	1,179,180 (72.2%)	69,971 (74.5%)	118,892 (80.3%)	112 (72.7%)	4,541,191 (76.9%)
Missing / Unknown	715,358 (11.6%)	106,029 (11.4%)	315,109 (8.8%)	149 (17.3%)	294,071 (18.0%)	10,438 (11.1%)	13,972 (9.4%)	34 (22.1%)	690,914 (11.7%)
Race									
Asian	182,864 (3.0%)	25,726 (2.8%)	109,531 (3.1%)	41 (4.8%)	47,566 (2.9%)	3,505 (3.7%)	6,106 (4.1%)	<20 (0%)	173,248 (2.9%)
Black or African American	874,647 (14.2%)	141,483 (15.2%)	539,304 (15.0%)	114 (13.2%)	193,746 (11.9%)	11,022 (11.7%)	16,221 (11.0%)	<20 (0%)	847,395 (14.3%)
Native Hawaiian / Pacific Islander	10,891 (0.2%)	1,827 (0.2%)	5,421 (0.2%)	<20 (0%)	3,642 (0.2%)	121 (0.1%)	253 (0.2%)	<20 (0%)	10,517 (0.2%)
White	3,861,223 (62.8%)	552,163 (59.4%)	2,359,258 (65.8%)	424 (49.1%)	949,378 (58.1%)	53,062 (56.5%)	96,524 (65.2%)	99 (64.3%)	3,711,538 (62.8%)
Other	1,045,699 (17.0%)	170,082 (18.3%)	481,042 (13.4%)	279 (32.3%)	394,296 (24.1%)	23,869 (25.4%)	27,168 (18.3%)	41 (26.6%)	994,621 (16.8%)
Missing / Unknown	170,316 (2.8%)	37,524 (4.0%)	87,474 (2.4%)	<20 (0%)	45,314 (2.8%)	2,220 (2.4%)	1,706 (1.2%)	<20 (0%)	166,390 (2.8%)

© 2022 Martin B et al. *JAMA Network Open.*

N3C Cohort Characteristics. Demographic characteristics for pediatric (A) and adult (B) N3C patients stratified by SARS-CoV-2 test type and result. Per N3C policy, cells with <20 patients are censored and replaced each with "<20 (0%)."
*Cell censored per N3C policy for having <20 associated patient encounters
Definitions: Ab = antibody, Ag = antigen, IQR = interquartile range, PCR = polymerase chain reaction, SD = standard deviation, UNK = Unknown

© 2022 Martin B et al. *JAMA Network Open.*

eTable 2: Adjusted Odds for Severe Disease by Demographic or Comorbidity Variable

Variable	OR (95% CI, p-value)	aOR (95% CI, p-value)
Sex (male vs. female)†	**1.37 (1.21-1.56, < 0.001)**	**1.32 (1.19-1.46, <0.001)**
Age†	0.88 (0.77-1.00, 0.05)	0.91 (0.82-1.02, 0.1)
Ethnicity†	0.96 (0.81-1.12, 0.58)	0.99 (0.89 – 1.09, 0.80)
Black or African American‡	**1.25 (1.06-1.47, 0.008)**	**1.29 (1.13-1.46, <0.001)**
Non-Black, Non-White	**1.23 (1.04-1.45, 0.01)**	1.08 (0.98-1.20, 0.13)
Obese (>= 95th percentile)	**1.19 (1.01-1.41, 0.04)**	**1.19 (1.00-1.41, 0.04)**
Asthma Diagnosis	0.91 (0.73-1.13, 0.38)	0.87 (0.72-1.05, 0.16)
PCCC		
Any Category	**1.20 (1.16-1.24, < 0.001)**	**1.16 (1.12–1.21, < 0.001)**
Congenital/Genetic	1.17 (0.91-1.49, 0.22)	1.10 (0.90-1.35, 0.35)
Cardiovascular	**1.76 (1.40-2.22, < 0.001)**	**1.57 (1.27-1.92, < 0.001)**
Gastrointestinal	1.03 (0.75-1.43, 0.85)	1.09 (0.78-1.52, 0.62)
Heme / Immune	0.83 (0.65-1.07, 0.16)	0.84 (0.63-1.10, 0.20)
Malignancy	**1.82 (1.33-2.49, <0.001)**	**1.70 (1.18-2.45, 0.005)**
Metabolic	1.16 (0.91-1.49, 0.23)	1.11 (0.95-1.30, 0.19)
Neonatal	0.99 (0.70-1.41, 0.95)	0.99 (0.71-1.37, 0.95)
Neuromuscular	**1.36 (1.06-1.74, 0.02)**	1.21 (0.87-1.69, 0.26)
Renal	**0.62 (0.45-0.86, 0.004)**	**0.59 (0.43-0.80, 0.001)**
Respiratory	**1.51 (1.10-2.08, 0.01)**	**1.46 (1.01-2.12, 0.04)**
Tech Dependence	**1.68 (1.19-2.38, 0.004)**	**1.49 (1.08-2.04, 0.01)**
Transplant	1.37 (0.82-2.27, 0.23)	1.21 (0.71-2.09, 0.48)

Sensitivity analysis evaluating impact of contributing health site on odds of severe disease.
A generalized estimating equation-based analysis to determine the adjusted odds of severe (versus moderate) disease for each demographic and comorbidity variable.
Abbreviations: aOR = adjusted odds ratio, CI = confidence interval, OR = odds ratio

© 2022 Martin B et al. *JAMA Network Open.*

† Odds ratio listed for sex is the odds of a male patient developing severe disease compared to a female patient; the odds ratio for age is the odds of a patient <12-years-old getting severe disease compared to a patient ≥12-years-old; the odds ratio for ethnicity is the odds of a Hispanic patient getting severe disease vs. a non-Hispanic patient (either not Hispanic or unknown)
‡ Odds ratio for a Black / African American child developing severe disease as compared to a White child
± Odds ratio for a non-Black, non-White child developing severe disease as compared to a White child
↡ BMI calculated as per the Centers for Disease Control and Prevention (CDC) guidelines[13] with obesity defined as any child ≥ 2-years-old with a BMI ≥ 95th percentile for age and sex. Percentages reported in the "Obese: BMI ≥95th" row represent the percent of patients with a known BMI value who had a BMI greater than 95th percentile for age and sex.

© 2022 Martin B et al. *JAMA Network Open.*

eTable 3. Medication Administration Rates by Pediatric Severity Subgroup

Medication Category	Medication Name	Severity Category	Children who received the medication, n (%)	p value*
Antimicrobial Overall		Moderate	2679 (30)	p < 0.001
		Severe	987 (69)	
Immunomodulation Overall		Moderate	1230 (14)	p < 0.001
		Severe	753 (53)	
Antimicrobial	Azithromycin	Moderate	272 (3)	p < 0.001
		Severe	89 (6)	
Antimicrobial	Remdesivir	Moderate	147 (2)	p < 0.001
		Severe	118 (8)	
Antimicrobial	Systemic Antibacterial	Moderate	2569 (29)	p < 0.001
		Severe	955 (67)	
Antimicrobial	Systemic Antifungal	Moderate	104 (1)	p < 0.001
		Severe	136 (10)	
Antimicrobial	Systemic Antiviral	Moderate	234 (3)	p < 0.001
		Severe	188 (13)	
Immunomodulation	Anakinra	Moderate	89 (1)	p < 0.001
		Severe	153 (11)	
Immunomodulation	Dexamethasone	Moderate	412 (5)	p < 0.001
		Severe	192 (13)	
Immunomodulation	Hydrocortisone	Moderate	133 (2)	p < 0.001
		Severe	255 (18)	
Immunomodulation	Infliximab	Moderate	52 (0.6)	p < 0.001
		Severe	39 (3)	
Immunomodulation	Methylprednisolone	Moderate	405 (5)	p < 0.001
		Severe	310 (22)	
Immunomodulation	Prednisone	Moderate	437 (5)	p < 0.001
		Severe	220 (15)	
Immunomodulation	Systemic Corticosteroid	Moderate	1142 (13)	p < 0.001
		Severe	684 (48)	

Statistical comparison of medication administration during pediatric hospitalization among moderate (N = 8,822) and severe (N = 1,423) subgroups.
*Comparison of the proportions of patients in the moderate vs severe subgroups who received a given medication / medication class.

© 2022 Martin B et al. *JAMA Network Open.*

eTable 4: Vital sign changes during the first week of hospitalization

Vital Sign	Severity Comparison Metric	Mean Value	Standard Error	CI Lower Limit	CI Upper Limit	p value
Systolic blood pressure (mmHg)	Severe: Day 0	103.2	0.8	101.6	104.8	
	Severe: Day 7	109.4	1.2	107.1	111.7	
	Severe: Day 7 - Day 0	6.2	1.4	3.4	9.0	p < 0.001
	Moderate: Day 0	108.4	0.3	107.8	109.1	
	Moderate: Day 7	112.0	0.8	110.3	113.6	
	Moderate: Day 7 - Day 0	3.5	1.0	1.6	5.5	p < 0.001
	Severe Day 0 - Moderate Day 0	-6.2	0.88	-7.92	-4.47	p < 0.001
Heart rate (beats per minute)	Severe: Day 0	115.2	1.3	112.7	117.7	
	Severe: Day 7	92.1	2.0	88.3	96.0	
	Severe: Day 7 - Day 0	-23.1	2.2	-27.4	-18.7	p < 0.001
	Moderate: Day 0	112.8	0.6	111.6	114.1	
	Moderate: Day 7	85.9	1.5	82.9	88.9	
	Moderate: Day 7 - Day 0	-26.9	1.7	-30.2	-23.5	p < 0.001
	Severe Day 0 - Moderate Day 0	4.69	1.52	1.72	7.66	p = 0.002
Respiratory rate (breaths per minute)	Severe: Day 0	27.1	0.5	26.1	28.2	
	Severe: Day 7	24.2	0.8	22.7	25.8	

© 2022 Martin B et al. *JAMA Network Open.*

	Severe: Day 7 - Day 0	-2.9	1.1	-5.0	-0.8	p = 0.006
	Moderate: Day 0	25.3	0.2	24.9	25.7	
	Moderate: Day 7	21.9	0.4	21.0	22.8	
	Moderate: Day 7 - Day 0	-3.4	0.5	-4.4	-2.4	p < 0.001
	Severe Day 0 - Moderate Day 0	2.05	0.5	1.06	3.04	p < 0.001
Diastolic blood pressure (mmHg)	Severe: Day 0	59.9	0.6	58.7	61.0	
	Severe: Day 7	62.9	0.9	61.0	64.7	
	Severe: Day 7 - Day 0	3.0	1.2	0.6	5.4	p = 0.01
	Moderate: Day 0	64.7	0.2	64.2	65.2	
	Moderate: Day 7	67.9	0.7	66.6	69.1	
	Moderate: Day 7 - Day 0	3.2	0.8	1.6	4.7	p < 0.001
	Severe Day 0 - Moderate Day 0	-5.1	0.65	-6.37	-3.83	p < 0.001
Mean arterial pressure (mmHg)	Severe: Day 0	70.4	1.4	67.7	73.1	
	Severe: Day 7	74.3	1.7	70.9	77.7	
	Severe: Day 7 - Day 0	3.9	1.9	0.2	7.7	p = 0.04
	Moderate: Day 0	74.3	0.7	72.9	75.8	
	Moderate: Day 7	82.8	1.6	79.6	86.0	
	Moderate: Day 7 - Day 0	8.4	2.0	4.5	12.4	p < 0.001

© 2022 Martin B et al. *JAMA Network Open.*

	Severe Day 0 - Moderate Day 0	-7.6	1.99	-11.5	-3.69	p < 0.001
Temperature (℃)	Severe: Day 0	37.2	0.0	37.1	37.3	
	Severe: Day 7	36.7	0.1	36.6	36.8	
	Severe: Day 7 - Day 0	-0.5	0.1	-0.7	-0.3	p < 0.001
	Moderate: Day 0	37.2	0.0	37.1	37.2	
	Moderate: Day 7	36.6	0.0	36.5	36.6	
	Moderate: Day 7 - Day 0	-0.6	0.0	-0.7	-0.5	p < 0.001
	Severe Day 0 - Moderate Day 0	-0.03	0.05	-0.13	0.07	p = 0.55
SpO2 (%)	Severe: Day 0	95.6	0.3	94.9	96.3	
	Severe: Day 7	95.1	0.5	94.1	96.0	
	Severe: Day 7 - Day 0	-0.6	0.6	-1.8	0.7	p = 0.37
	Moderate: Day 0	97.7	0.1	97.5	97.9	
	Moderate: Day 7	97.6	0.2	97.2	97.9	
	Moderate: Day 7 - Day 0	-0.1	0.2	-0.5	0.3	p = 0.50
	Severe Day 0 - Moderate Day 0	-2.63	0.43	-3.48	-1.78	p < 0.001

Statistical comparison of the initial values and trends (from day 0 to day 7 of hospitalization) for vital signs between moderate and severe pediatric subgroups.

Abbreviations: CI = confidence interval, SpO2 = peripheral oxygen saturation

© 2022 Martin B et al. *JAMA Network Open.*

eTable 5: Changes in laboratory test values during the first week of hospitalization

Laboratory Test	Severity Comparison Metric	Mean	Standard Error	CI Lower	CI Upper	p value
Albumin (g/dL)	Severe Day 7 - Day 0	-0.2	0.1	-0.3	-0.1	p < 0.001
	Moderate Day 7 - Day 0	-0.6	0.0	-0.7	-0.6	p < 0.001
	Severe Day 0 - Moderate Day 0	-0.54	0.03	-0.61	-0.47	p < 0.001
ALT (SGPT), IU/L	Severe Day 7 - Day 0	-99.0	55.4	-207.6	9.6	p = 0.07
	Moderate Day 7 - Day 0	-132.7	85.7	-300.6	35.3	p = 0.12
	Severe Day 0 - Moderate Day 0	33.65	13.98	6.24	61.06	p = 0.02
AST (SGOT), IU/L	Severe Day 7 - Day 0	-163.1	57.9	-276.5	-49.6	p = 0.005
	Moderate Day 7 - Day 0	-93.0	43.9	-179.0	-7.0	p = 0.03
	Severe Day 0 - Moderate Day 0	48.46	16.78	15.57	81.36	p = 0.004
Bilirubin (Unconjugated / Indirect), mg/dL	Severe Day 7 - Day 0	0.0	0.2	-0.5	0.4	p = 0.85
	Moderate Day 7 - Day 0	-0.3	0.3	-0.8	0.2	p = 0.26
	Severe Day 0 - Moderate Day 0	-0.05	0.17	-0.38	0.28	p = 0.77
Bilirubin (Conjugated / Direct),mg/dL	Severe Day 7 - Day 0	-0.2	0.2	-0.5	0.2	p = 0.33
	Moderate Day 7 - Day 0	-0.6	0.2	-1.0	-0.2	p = 0.003
	Severe Day 0 - Moderate Day 0	0.09	0.07	-0.04	0.22	p = 0.18
Bilirubin (total), mg/dL	Severe Day 7 - Day 0	-0.1	0.3	-0.6	0.4	p = 0.67
	Moderate Day 7 - Day 0	-0.6	0.2	-0.9	-0.3	p < 0.001
	Severe Day 0 - Moderate Day 0	0.11	0.09	-0.07	0.29	p = 0.21
BNP (all forms), pg/mL	Severe Day 7 - Day 0	-4714.9	802.1	-6286.9	-3142.9	p < 0.001
	Moderate Day 7 - Day 0	-806.3	337.0	-1466.8	-145.8	p = 0.02
	Severe Day 0 - Moderate Day 0	3,108.1	687.7	1,760.1	4,456.0	p < 0.001
BUN, mg/dL	Severe Day 7 - Day 0	-0.3	2.0	-4.2	3.7	p = 0.90
	Moderate Day 7 - Day 0	-3.4	0.9	-5.1	-1.6	p < 0.001
	Severe Day 0 - Moderate Day 0	4.65	0.66	3.35	5.94	p < 0.001
BUN/Creatinine ratio	Severe Day 7 - Day 0	12.5	2.6	7.5	17.5	p < 0.001
	Moderate Day 7 - Day 0	7.7	2.0	3.7	11.7	p < 0.001
	Severe Day 0 - Moderate Day 0	2.99	2.10	-1.13	7.10	p = 0.15
c-reactive protein (CRP), mg/L	Severe Day 7 - Day 0	-137.0	10.7	-157.9	-116.0	p < 0.001
	Moderate Day 7 - Day 0	-93.4	6.8	-106.8	-80.0	p < 0.001
	Severe Day 0 - Moderate Day 0	55.98	6.77	42.72	69.24	p < 0.001
Chloride, mmol/L	Severe Day 7 - Day 0	-2.2	0.7	-3.7	-0.8	p = 0.003
	Moderate Day 7 - Day 0	0.5	0.4	-0.4	1.3	p = 0.27
	Severe Day 0 - Moderate Day 0	-0.18	0.25	-0.67	0.31	p = 0.48
Creatinine, mg/dL	Severe Day 7 - Day 0	-0.4	0.1	-0.6	-0.2	p < 0.001
	Moderate Day 7 - Day 0	-0.4	0.1	-0.6	-0.2	p < 0.001
	Severe Day 0 - Moderate Day 0	0.22	0.06	0.12	0.33	p < 0.001
D-Dimer, mg/L	Severe Day 7 - Day 0	0.4	0.7	-1.0	1.8	p = 0.55
	Moderate Day 7 - Day 0	-0.6	0.3	-1.2	0.0	p = 0.06
	Severe Day 0 - Moderate Day 0	1.22	0.37	0.49	1.95	p = 0.001
Erythrocyte Sed. Rate, mm/hr	Severe Day 7 - Day 0	3.2	4.8	-6.2	12.7	p = 0.50
	Moderate Day 7 - Day 0	12.0	4.0	4.1	19.9	p = 0.003
	Severe Day 0 - Moderate Day 0	5.68	2.11	1.54	9.82	p = 0.007
Ferritin, ng/mL	Severe Day 7 - Day 0	-542.5	153.1	-842.6	-242.4	p < 0.001
	Moderate Day 7 - Day 0	-98.2	149.5	-391.1	194.7	p = 0.51
	Severe Day 0 - Moderate Day 0	232.82	102.58	31.76	433.88	p = 0.02
Fibrinogen	Severe Day 7 - Day 0	-141.5	24.6	-189.7	-93.2	p < 0.001
	Moderate Day 7 - Day 0	-163.1	24.4	-210.9	-115.3	p < 0.001
	Severe Day 0 - Moderate Day 0	-13.67	16.95	-46.90	19.56	p = 0.42

© 2022 Martin B et al. *JAMA Network Open.*

Glucose, mg/dL	Severe Day 7 - Day 0	-19.7	5.4	-30.4	-9.1	p < 0.001
	Moderate Day 7 - Day 0	-43.0	5.7	-54.1	-31.9	p < 0.001
	Severe Day 0 - Moderate Day 0	14.24	2.64	9.07	19.41	p < 0.001
Hemoglobin, g/dL	Severe Day 7 - Day 0	-0.7	0.2	-1.1	-0.4	p < 0.001
	Moderate Day 7 - Day 0	-1.4	0.1	-1.7	-1.2	p < 0.001
	Severe Day 0 - Moderate Day 0	-0.53	0.10	-0.73	-0.33	p < 0.001
IL-6, pg/mL	Severe Day 7 - Day 0	12.4	87.4	-158.9	183.7	p = 0.89
	Moderate Day 7 - Day 0	0.0	46.9	-91.9	91.9	p = 1.00
	Severe Day 0 - Moderate Day 0	78.89	27.77	24.47	133.32	p = 0.005
Lactate Dehydrogenase (LDH), units/L	Severe Day 7 - Day 0	-276.5	143.0	-556.9	3.8	p = 0.05
	Moderate Day 7 - Day 0	-160.9	64.8	-287.8	-33.9	p = 0.01
	Severe Day 0 - Moderate Day 0	159.38	57.40	46.88	271.88	p = 0.006
Lactate, mM	Severe Day 7 - Day 0	-2.1	0.3	-2.7	-1.5	p < 0.001
	Moderate Day 7 - Day 0	-2.9	1.2	-5.2	-0.6	p = 0.02
	Severe Day 0 - Moderate Day 0	0.49	0.11	0.27	0.71	p < 0.001
Lymphocytes (absolute), x10^3/μL	Severe Day 7 - Day 0	-0.1	0.2	-0.5	0.3	p = 0.64
	Moderate Day 7 - Day 0	0.3	0.2	-0.1	0.6	p = 0.12
	Severe Day 0 - Moderate Day 0	-0.27	0.10	-0.48	-0.07	p = 0.009
Lymphocytes (relative), %	Severe Day 7 - Day 0	3.5	1.1	1.4	5.6	p < 0.001
	Moderate Day 7 - Day 0	10.5	0.8	8.8	12.1	p < 0.001
	Severe Day 0 - Moderate Day 0	-7.40	0.78	-8.92	-5.87	p < 0.001
Neutrophils (absolute), x10^3/μL	Severe Day 7 - Day 0	-0.2	0.8	-1.7	1.3	p = 0.82
	Moderate Day 7 - Day 0	-1.5	0.4	-2.2	-0.8	p < 0.001
	Severe Day 0 - Moderate Day 0	1.98	0.34	1.31	2.66	p < 0.001
Neutrophils (relative), %	Severe Day 7 - Day 0	-3.2	1.6	-6.4	0.0	p = 0.05
	Moderate Day 7 - Day 0	-8.6	1.2	-10.9	-6.2	p < 0.001
	Severe Day 0 - Moderate Day 0	6.72	1.23	4.31	9.13	p < 0.001
NT-proBNP, pg/mL	Severe Day 7 - Day 0	-9538.9	1765.5	-12999.2	-6078.6	p < 0.001
	Moderate Day 7 - Day 0	-1178.9	523.1	-2204.2	-153.7	p = 0.02
	Severe Day 0 - Moderate Day 0	6,297	1,479	3,399	9,196	p < 0.001
pH	Severe Day 7 - Day 0	0.10	0.01	0.08	0.12	p < 0.001
	Moderate Day 7 - Day 0	0.15	0.02	0.11	0.18	p < 0.001
	Severe Day 0 - Moderate Day 0	-0.01	0.01	-0.02	0.01	p = 0.48
Platelet count, x10^3/μL	Severe Day 7 - Day 0	69.9	10.2	49.8	89.9	p < 0.001
	Moderate Day 7 - Day 0	80.7	7.2	66.6	94.9	p < 0.001
	Severe Day 0 - Moderate Day 0	-48.59	5.41	-59.20	-37.98	p < 0.001
Potassium, mmol/L	Severe Day 7 - Day 0	0.03	0.06	-0.09	0.14	p = 0.64
	Moderate Day 7 - Day 0	-0.09	0.04	-0.17	-0.01	p = 0.03
	Severe Day 0 - Moderate Day 0	-0.17	0.03	-0.23	-0.11	p < 0.001
Procalcitonin, ng/mL	Severe Day 7 - Day 0	-11.9	2.6	-16.9	-6.8	p < 0.001
	Moderate Day 7 - Day 0	-6.0	1.9	-9.8	-2.2	p = 0.002
	Severe Day 0 - Moderate Day 0	8.28	2.65	3.08	13.48	p = 0.002
Sodium, mmol/L	Severe Day 7 - Day 0	1.6	0.7	0.2	3.0	p = 0.03
	Moderate Day 7 - Day 0	1.5	0.4	0.6	2.4	p = 0.001
	Severe Day 0 - Moderate Day 0	-0.88	0.22	-1.32	-0.45	p < 0.001
Troponin all types, ng/mL	Severe Day 7 - Day 0	-1.64	0.57	-2.76	-0.52	p = 0.004
	Moderate Day 7 - Day 0	-1.30	0.49	-2.26	-0.35	p = 0.008
	Severe Day 0 - Moderate Day 0	0.45	0.21	0.03	0.87	p = 0.04
White blood cell count, x10^3/μL	Severe Day 7 - Day 0	-2.81	2.81	-8.33	2.70	p = 0.32
	Moderate Day 7 - Day 0	-2.00	1.09	-4.14	0.13	p = 0.07
	Severe Day 0 - Moderate Day 0	2.42	0.81	0.84	4.00	p = 0.003

Statistical comparison of the initial values and trends (from day 0 to day 7 of hospitalization) for laboratory results between moderate and severe pediatric subgroups. Day 7 and day 0 mean values not shown.

© 2022 Martin B et al. *JAMA Network Open.*

Abbreviations: ALT = alanine aminotransferase, AST = aspartate aminotransferase, BNP = brain natriuretic peptide, BUN = blood urea nitrogen, CI = confidence interval, IL-6 interleukin-6, NT-proBNP = N-terminal pro brain natriuretic peptide.

© 2022 Martin B et al. *JAMA Network Open.*

eTable 6: Proportion of children with a given lab test result by severity subgroup

Lab Test	Severity Category	Children with Lab Available, n (%)	Adults with Lab Available, n (%)
ALT (SGPT), IU/L	Severe	946 (66)	32201 (78)
	Moderate	3471 (39)	91101 (67)
AST (SGOT), IU/L	Severe	1002 (70)	33892 (82)
	Moderate	3789 (43)	103552 (76)
Albumin (g/dL)	Severe	1013 (71)	34216 (83)
	Moderate	4042 (46)	107634 (79)
BNP (all forms), pg/mL	Severe	554 (39)	22098 (53)
	Moderate	1276 (14)	49156 (36)
BUN, mg/dL	Severe	1131 (79)	37629 (91)
	Moderate	4967 (56)	122020 (90)
BUN/Creatinine ratio	Severe	178 (13)	11721 (28)
	Moderate	1245 (14)	42383 (31)
Bilirubin (Conjugated/Direct), mg/dL	Severe	599 (42)	21539 (52)
	Moderate	1651 (19)	53646 (39)
Bilirubin (total), mg/dL	Severe	1032 (73)	35923 (87)
	Moderate	4067 (46)	111413 (82)
Bilirubin - (Unconjugated/Indirect), mg/dL	Severe	278 (20)	6432 (16)
	Moderate	527 (6)	14065 (10)
Chloride, mmol/L	Severe	1142 (80)	37616 (91)
	Moderate	4987 (57)	121951 (90)
Creatinine, mg/dL	Severe	1133 (80)	37667 (91)
	Moderate	4984 (56)	122251 (90)
D-Dimer, mg/L FEU	Severe	380 (27)	19825 (48)
	Moderate	1115 (13)	58530 (43)
Erythrocyte Sed. Rate, mm/hr	Severe	570 (40)	11167 (27)
	Moderate	1554 (18)	24466 (18)
Ferritin, ng/mL	Severe	588 (41)	24927 (60)
	Moderate	1400 (16)	69839 (51)
Fibrinogen mg/dL	Severe	617 (43)	11966 (29)
	Moderate	833 (9)	15464 (11)
Glucose, mg/dL	Severe	1134 (80)	37510 (90)
	Moderate	5060 (57)	121217 (89)
Hemoglobin, g/dL	Severe	1152 (81)	37566 (91)
	Moderate	5195 (59)	123850 (91)
IL-6, pg/mL	Severe	137 (10)	3790 (9)

© 2022 Martin B et al. *JAMA Network Open.*

	Moderate	111 (1)	6042 (4)
Lactate Dehydrogenase (LDH), units/L	Severe	398 (28)	19753 (48)
	Moderate	788 (9)	53493 (39)
Lactate, mmol/L	Severe	815 (57)	27913 (67)
	Moderate	1399 (16)	57520 (42)
Lymphocytes (absolute), x$10^3/\mu$L	Severe	883 (62)	33569 (81)
	Moderate	4114 (47)	109304 (80)
Lymphocytes (relative), %	Severe	1045 (73)	33268 (80)
	Moderate	4677 (53)	112995 (83)
NT pro BNP, pg/mL	Severe	309 (22)	11608 (28)
	Moderate	889 (10)	29859 (22)
Neutrophils (absolute), x$10^3/\mu$L	Severe	757 (53)	32152 (78)
	Moderate	3684 (42)	102215 (75)
Neutrophils (relative), %	Severe	671 (47)	33268 (80)
	Moderate	3224 (37)	94513 (69)
Platelet count, x$10^3/\mu$L	Severe	928 (65)	36277 (88)
	Moderate	4339 (49)	116436 (86)
Potassium, mmol/L	Severe	1143 (80)	37619 (91)
	Moderate	4956 (56)	121795 (90)
Procalcitonin, ng/mL	Severe	325 (23)	17444 (42)
	Moderate	940 (11)	43524 (32)
Sodium, mmol/L	Severe	1144 (80)	37628 (91)
	Moderate	4999 (57)	121500 (89)
Troponin all types, ng/mL	Severe	468 (33)	16632 (40)
	Moderate	726 (8)	35537 (26)
White blood cell count, x$10^3/\mu$L	Severe	893 (63)	36499 (88)
	Moderate	4397 (50)	120301 (88)
c-reactive protein CRP, mg/L	Severe	702 (49)	20175 (49)
	Moderate	1655 (19)	49491 (36)
pH	Severe	730 (51)	26237 (63)
	Moderate	1015 (12)	32237 (24)

Proportion of children in the moderate (N = 8,822) and severe (N = 1,423) disease subgroups alongside the number and percent of adults in the moderate (N = 136,055) and severe (N = 41,457) disease subgroups who had at least one value for a given lab test available during their inpatient hospital encounter.

eTable 7: MIS-C versus acute COVID-19 characteristics, outcomes, and risk factors.

Category	MIS-C (N = 707), n (%)	Acute COVID-19 (N = 8,241), n(%)	MIS-C vs. COVID-19 OR (95% CI, p-value)	MIS-C vs. COVID-19 aOR (95% CI, p-value)
Demographics				
**Age**[†]			**1.81 (1.51-2.18, <0.001)**	**1.70 (1.14-2.55, 0.01)**
Age < 1 year				
Age 1-5 years				
Age 5-12 years				
Age 12-18 years				
**Sex**[†]			**1.59 (1.33-1.90, <0.001)**	**1.64 (1.33-2.02, <0.001)**
Male	416 (59)	3987 (48)		
Female	291 (41)	4237 (51)		
**Race**[±]			**0.65 (0.51-0.83, <0.001)**	**0.80 (0.65-0.98, 0.03)**
Asian	<20 (0)	168 (2)		
Black[‡]	250 (35)	2065 (25)	**1.44 (1.17-1.77, <0.001)**	**1.32 (1.03-1.69, 0.03)**
White	277 (39)	3578 (43)		
Other Race	39 (6)	293 (4)		
**Ethnicity**[†]			1.16 (0.92-1.46, 0.22)	1.23 (0.97-1.55, 0.09)
Hispanic	153 (22)	2155 (26)		
Not Hispanic	413 (58)	5034 (61)		
No Matching Ethnicity	138 (20)	1015 (12)		
Preselected Comorbidities				
Asthma	47 (7)	850 (10)	**0.69 (0.48-1.00, 0.05)**	**0.66 (0.49-0.90, 0.008)**
Diabetes	<20 (0)	256 (3)	*	*
Obesity[ⱡ]	169 (32)	1275 (33)	**1.76 (1.40-2.22, <0.001)**	**1.76 (1.40-2.22, < 0.001)**
Pediatric Complex Chronic Conditions				
Any PCCC Category	112 (16)	2208 (27)	**0.72 (0.65-0.80, <0.001)**	**0.75 (0.62-0.90, 0.002)**
Cong Genetic PCCC	25 (4)	659 (8)	0.69 (0.41-1.14, 0.15)	0.61 (0.29-1.28, 0.19)
CV PCCC	52 (7)	777 (9)	1.42 (0.93-2.16, 0.10)	1.38 (0.95-2.00, 0.09)
GI PCCC	22 (3)	650 (8)	1.07 (0.55-2.09, 0.85)	1.03 (0.43-2.46, 0.94)

© 2022 Martin B et al. *JAMA Network Open.*

Heme Immune PCCC	43 (6)	737 (9)	0.65 (0.41-1.05, 0.08)	0.62 (0.36-1.05,0.08)
Malignancy PCCC	<20 (0)	333 (4)	**0.33 (0.13-0.86, 0.02)**	**0.32 (0.12-0.84, 0.02)**
Metabolic PCCC	41 (6)	700 (9)	**0.56 (0.32-0.98, 0.04)**	**0.47 (0.25-0.91, 0.02)**
Neonatal PCCC	<20 (0)	264 (3)	**0.36 (0.14-0.92, 0.03)**	0.26 (0.06-1.21, 0.09)
Neuromuscular PCCC	<20 (0)	721 (9)	**0.28 (0.14-0.57, < 0.001)**	**0.25 (0.11-0.57, 0.001)**
Renal PCCC	20 (3)	445 (5)	1.38 (0.74-2.56, 0.31)	1.46 (0.74-2.86, 0.27)
Respiratory PCCC	<20 (0)	425 (5)	0.70 (0.32-1.50, 0.35)	0.75 (0.29-1.95, 0.55)
COVID Testing				
COVID +PCR/Ag	207 (29)	8241 (100)		
COVID +Antibody	624 (88)	<20 (0)		
Laboratory Test				
ESR (High)	509 (72)	693 (8)		
Albumin (Low)	454 (64)	648 (8)		
LDH (High)	436 (62)	516 (6)		
Serum Sodium (Low)	426 (60)	1075 (13)		
D-dimer (High)	401 (57)	591 (7)		
NT Pro BNP (High)	363 (51)	315 (4)		
ALC (Low)	351 (50)	1219 (15)		
CRP (High)	349 (49)	343 (4)		
Ferritin (High)	285 (40)	285 (4)		
Platelet Count (Low)	261 (37)	668 (8)		
WBC (High)	229 (32)	829 (10)		
ALT (High)	217 (31)	658 (8)		
Procalcitonin (High)	201 (28)	227 (3)		
BNP (High)	184 (26)	80 (1)		
IL6 (High)	137 (19)	107 (1)		
WBC (Low)	122 (17)	840 (10)		
Hgb (Low)	59 (8)	342 (4)		
Fibrinogen (Low)	31 (4)	63 (1)		

© 2022 Martin B et al. *JAMA Network Open.*

Clinical Outcomes				
Invasive Mechanical Ventilation[¥]	117 (17)	514 (6)		
ECMO	<20 (0)	25 (0.3)		
Vasopressor / Inotropic Support[¥]	191 (27)	426 (5)		
Deaths	<20 (0)	95 (1)		

Number and percent of children in the MIS-C and acute COVID-19 subgroups with a given demographic characteristic, pre-existing comorbidity, abnormal lab value during hospitalization, or clinical outcome. The odds ratio (OR) and adjusted odds ratio (aOR) (controlling for healthcare site) for having MIS-C (versus acute COVID-19) is shows for each. Those with a p-value < 0.05 are shown in bold. Given the low number of patients with diabetes mellitus, calculation of OR's for this potential risk factor was not feasible.

Abbreviations: ALC = absolute lymphocyte count, ANC = absolute neutrophil count, CRP = c-reactive protein, hgb = hemoglobin, IL6 = interleukin-6

* Given the low number of children with diabetes mellitus in the subgroups, odds ratios for diabetes mellitus were not able to be calculated

† Odds ratio listed for sex is the odds of a male patient developing severe disease compared to a female patient; the odds ratio for age is the odds of a patient <12-years-old getting severe disease compared to a patient ≥12-years-old; the odds ratio for ethnicity is the odds of a Hispanic patient getting severe disease vs. a non-Hispanic patient (either not Hispanic or unknown)

‡ Odds ratio for a Black / African American child developing severe disease as compared to a White child

± Odds ratio for a non-Black, non-White child developing severe disease as compared to a White child. Missing / Unknown and Native Hawaiian / Pacific Islander racial categories not shown.

ǂ BMI calculated as per the Centers for Disease Control and Prevention (CDC) guidelines[13] with obesity defined as any child ≥ 2-years-old with a BMI ≥ 95th percentile for age and sex. The percent of children with obesity was calculated by dividing the number of children ≥2-years-old who had a BMI for age and sex ≥95th percentile in that subgroup, by the number of children in that subgroup who were ≥2 years old and had a BMI measurement available (N = 529 for MIS-C and N = 3,829 for acute COVID-19).

¥ p < 0.001 for comparison of subgroup proportions

© 2022 Martin B et al. *JAMA Network Open.*

eTable 8: Delta versus pre-Delta differences in patient characteristics, outcomes, and severity risk factors.

	**Pre-Delta Era**		**Delta Era**		**Odds for Severe Disease**	
**Variable**	**All Cases (N = 138,071)**	**Hospitalized (N = 8,507)**	**All Cases (N = 29,191)**	**Hospitalized (N = 1,738)**	**OR Change (95% CI, p-value)**	**aOR Change (95% CI, p-value)**
Sex†					1.36 (0.92-2.01, 0.12)	1.45 (1.13-1.87, 0.003)
Male	69,062 (50.0%)	4,279 (50.3%)	14,727 (50.5%)	830 (47.8%)		
Female	68,442 (49.6%)	4,213 (49.5%)	14,440 (49.5%)	906 (52.1%)		
Other	567 (0.4%)	<20 (0%)	24 (0.1%)	<20 (0%)		
Age†						
Age in years, median (IQR)	12.3 (6.2, 16.4)	11.1 (2.7, 16.0)	10.3 (5.3, 14.9)	9.5 (1.9, 15.4)	1.14 (0.77-1.71, 0.51)	1.13 (0.91-1.40, 0.26)
Ethnicity†					0.56 (0.31-0.99, 0.05)	0.67 (0.44-1.02, 0.06)
Hispanic or Latino	31,248 (22.6%)	2,401 (28.2%)	5,220 (17.9%)	287 (16.5%)		
Not Hispanic or Latino	85,112 (61.6%)	4,854 (57.1%)	20,119 (68.9%)	1,262 (72.6%)		
Missing/Unknown	21,711 (15.7%)	1,252 (14.7%)	3,852 (13.2%)	189 (10.9%)		
Race±					1.69 (1.02-2.82, 0.04)	1.58 (1.09-2.30, 0.02)
Asian	3,133 (2.3%)	194 (2.3%)	475 (1.6%)	21 (1.2%)		
Black or African American‡	20,171 (14.6%)	2,096 (24.6%)	6,859 (23.5%)	611 (35.2%)	0.73 (0.45-1.18, 0.20)	0.90 (0.64-1.25, 0.52)
Missing/Unknown	3,467 (2.5%)	301 (3.5%)	631 (2.2%)	22 (1.3%)		
Native Hawaiian or Other Pacific Islander	352 (0.3%)	31 (0.4%)	42 (0.1%)	<20 (0%)		
White	77,211 (55.9%)	3,580 (42.1%)	15,636 (53.6%)	753 (43.3%)		
Other	33,623 (24.4%)	2,289 (26.9%)	5,520 (18.9%)	323 (18.6%)		
Comorbidity						
Known BMI	57,481 (41.6%)	4,356 (51.2%)	12,398 (42.5%)	765 (44.0%)		

© 2022 Martin B et al. *JAMA Network Open.*

Obese (≥95th percentile)[ɫ]	14,477 (25.2%)	1,402 (32.2%)	3,327 (26.8%)	254 (33.2%)	0.77 (0.45-1.31, 0.33)	0.77 (0.44-1.33, 0.35)
Asthma	10,583 (7.7%)	801 (9.4%)	2,706 (9.3%)	198 (11.4%)	1.43 (0.80-2.57, 0.23)	1.35 (0.79-2.31, 0.28)
Diabetes	938 (0.7%)	253 (3.0%)	133 (0.5%)	32 (1.8%)	*	*
PCCC Category						
Any PCCC	19468 (14%)	2205 (26%)	4318 (15%)	432 (25%)	1.04 (0.95-1.13, 0.38)	1.04 (0.98-1.11, 0.20)
Congenital / Genetic	5430 (4%)	625 (7%)	1246 (4%)	151 (9%)	0.77 (0.39-1.53, 0.45)	0.86 (0.48-1.54, 0.61)
Cardiovascular	4040 (3%)	803 (9%)	760 (3%)	154 (9%)	1.31 (0.68-2.51, 0.42)	1.38 (0.84-2.27, 0.21)
Gastrointestinal	2982 (2%)	649 (8%)	556 (2%)	119 (7%)	0.95 (0.39-2.33, 0.91)	0.95 (0.36-2.47, 0.91)
Heme/Immune	4605 (3%)	720 (8%)	1290 (4%)	162 (9%)	1.76 (0.89-3.46, 0.10)	1.64 (1.15-2.31, 0.006)
Malignancy	1469 (1%)	344 (4%)	253 (1%)	48 (3%)	1.43 (0.51-4.02, 0.50)	1.35 (0.54-3.37, 0.52)
Metabolic	4549 (3%)	694 (8%)	964 (3%)	134 (8%)	1.17 (0.57-2.38, 0.67)	1.38 (0.83-2.30, 0.22)
Neonatal	1466 (1%)	234 (3%)	384 (1%)	64 (4%)	1.18 (0.46-3.06, 0.73)	1.18 (0.62-2.25, 0.62)
Neuromuscular	3532 (3%)	709 (8%)	717 (2%)	143 (8%)	1.54 (0.76-3.15, 0.23)	1.64 (0.69-3.09, 0.32)
Renal	2330 (2%)	446 (5%)	458 (2%)	88 (5%)	0.55 (0.20-1.54, 0.26)	0.61 (0.21-1.82, 0.38)
Respiratory	2139 (2%)	419 (5%)	419 (1%)	80 (5%)	0.66 (0.26-1.70, 0.39)	0.63 (0.23-1.72, 0.37)
Tech Dependence	1830 (1%)	616 (7%)	358 (1%)	126 (7%)	0.74 (0.27-2.03, 0.56)	0.71 (0.29-1.77, 0.47)
Transplant	222 (0%)	94 (1%)	32 (0.1%)	<20 (0%)	1.37 (0.24-7.71, 0.72)	1.27 (0.35-4.61, 0.72)
Clinical Outcomes						
Mechanical Ventilation	700 (0.5%)	700 (8.2%)	96 (0.3%)	96 (5.5%)		
Vasoactive-Inotropic Support	760 (0.6%)	760 (8.9%)	108 (0.4%)	108 (6.2%)		
ECMO	39 (0.03%)	39 (0.5%)	<20 (0%)	<20 (0%)		
Mortality	134 (0.1%)	122 (1.4%)	<20 (0%)	<20 (0%)		

© 2022 Martin B et al. *JAMA Network Open.*

The number and percent of children in each era (Delta, defined as patients with a visit start date on/after 6/26/2021, and pre-Delta, defined as those with a start date before 6/26/2021) with a given demographic characteristic, pre-existing comorbidity, or clinical outcome. Also shown is the change in the odds ratios (ORs) for severe (as compared to moderate) disease observed during the Delta era as compared with the pre-Delta era. Similarly, the change in adjusted odds ratio (aOR) (correcting for health care site), is also shown.
*Given the low number of children with diabetes mellitus, calculation of OR's for this potential risk factor was not feasible
† Odds ratio listed for sex is the odds of a male patient developing severe disease compared to a female patient; the odds ratio for age is the odds of a patient <12-years-old getting severe disease compared to a patient ≥12-years-old; the odds ratio for ethnicity is the odds of a Hispanic patient getting severe disease vs. a non-Hispanic patient (either not Hispanic or unknown)
‡ Odds ratio for a Black / African American child developing severe disease as compared to a White child
± Odds ratio for a non-Black, non-White child developing severe disease as compared to a White child
↡ BMI calculated as per the Centers for Disease Control and Prevention (CDC) guidelines[13] with obesity defined as any child ≥ 2-years-old with a BMI ≥ 95th percentile for age and sex. The percent of children with obesity was calculated by dividing the number of children ≥2-years-old who had a BMI for age and sex ≥95th percentile in that subgroup, by the number of children in that subgroup who were ≥2 years old and had a BMI measurement available.

eFigure 1: Geographic distribution and case incidence over time N3C pediatric patients.

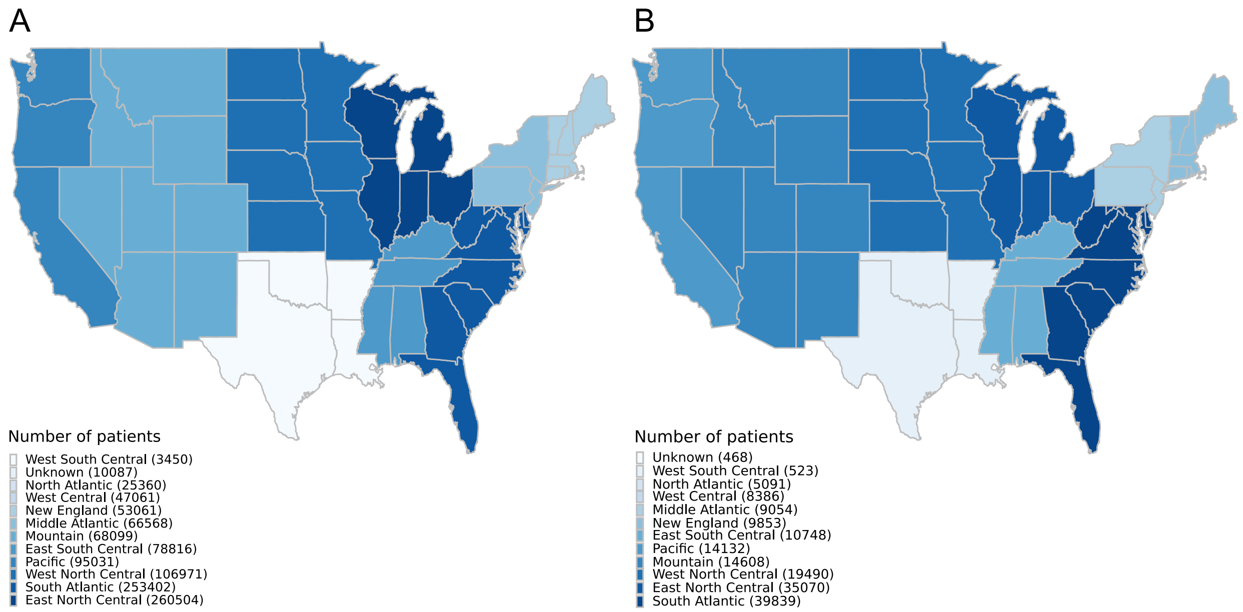

(a) Geographic distribution of all pediatric N3C patients (N = 1,068,410). (b) Geographic distribution of SARS-CoV-2 positive pediatric cases only (N = 167,262).

eFigure 2: Subregion positive test result trends by test type

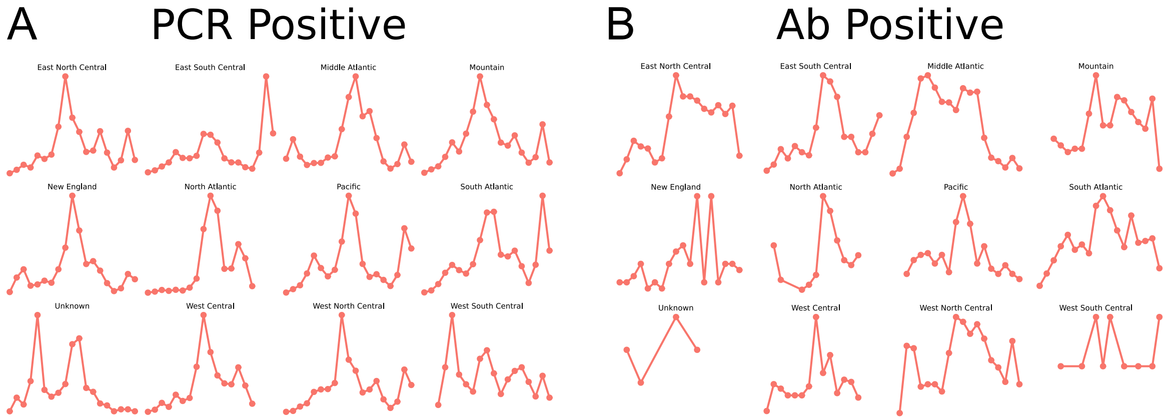

(a) Monthly trends for positive PCR/Ag pediatric SARS-CoV-2 tests by subregion. (b) Monthly trends for positive SARS-CoV-2 antibody tests by subregion. Note that these individual subregion plots are scaled such that the maximum value is located at the top of the chart regardless of absolute value.

© 2022 Martin B et al. *JAMA Network Open.*

eFigure 3: Change in qualifying criteria for “severe” subgroup classification over time

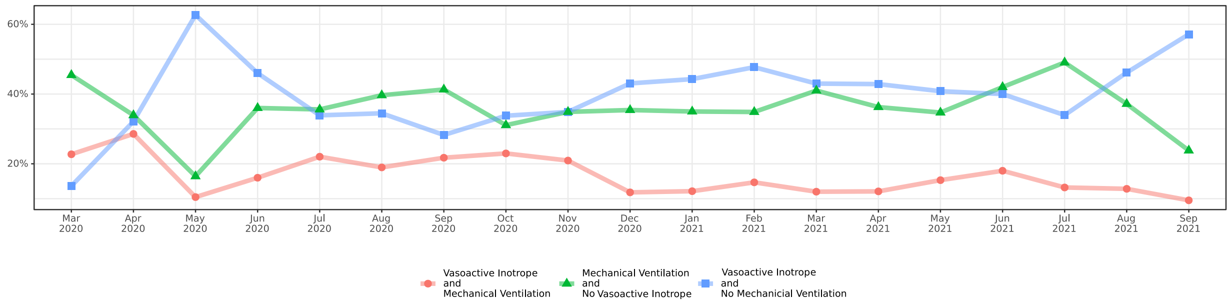

Shown here is the percent of patient encounters each month who meet different criteria for “severe” maximum clinical severity status during the study period.

© 2022 Martin B et al. *JAMA Network Open.*

eFigure 4: Maximum clinical severity distributions among prespecified age groups.

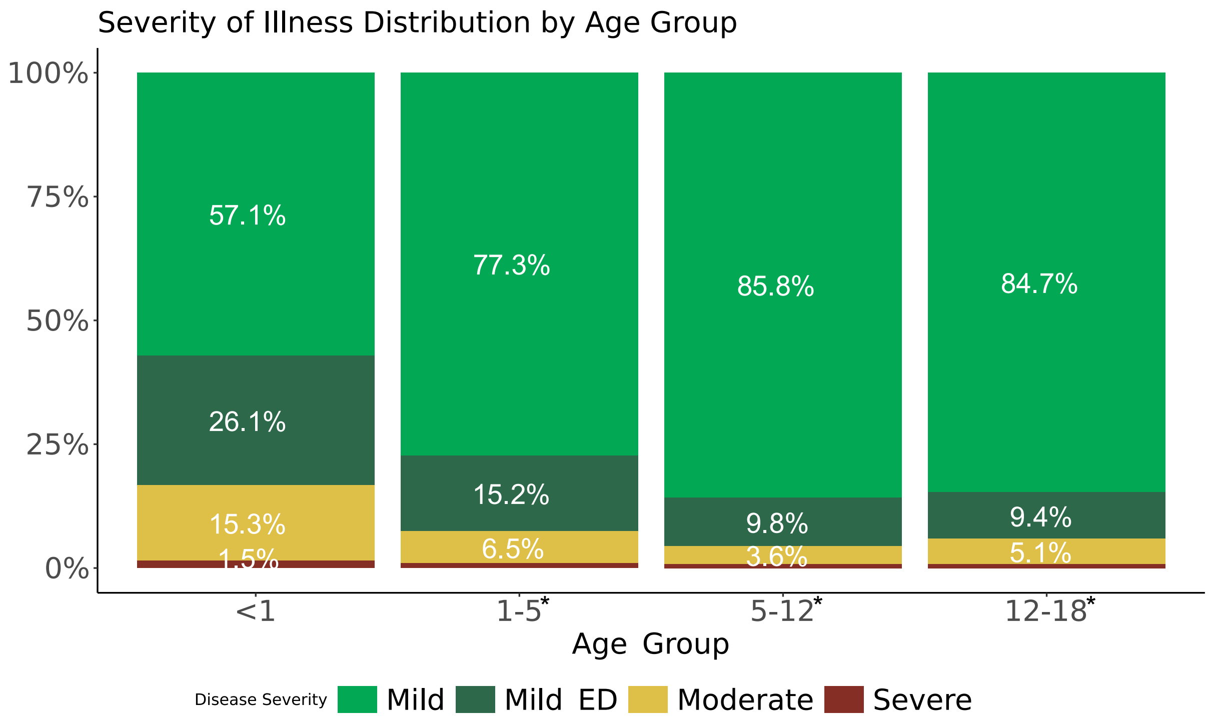

For each specified age group (<1 year old, 1-5 years old, 5-12 years old, and 12-18 years old) the percent of patients within each of the four maximum clinical severity categories is shown.

*Severe category percentages for the 1-5, 5-12, and 12-18 year old age subgroups are 1%, 0.8%, and 0.8%, respectively.

© 2022 Martin B et al. *JAMA Network Open.*

eFigure 5: Antimicrobial and immunomodulatory medication usage trends

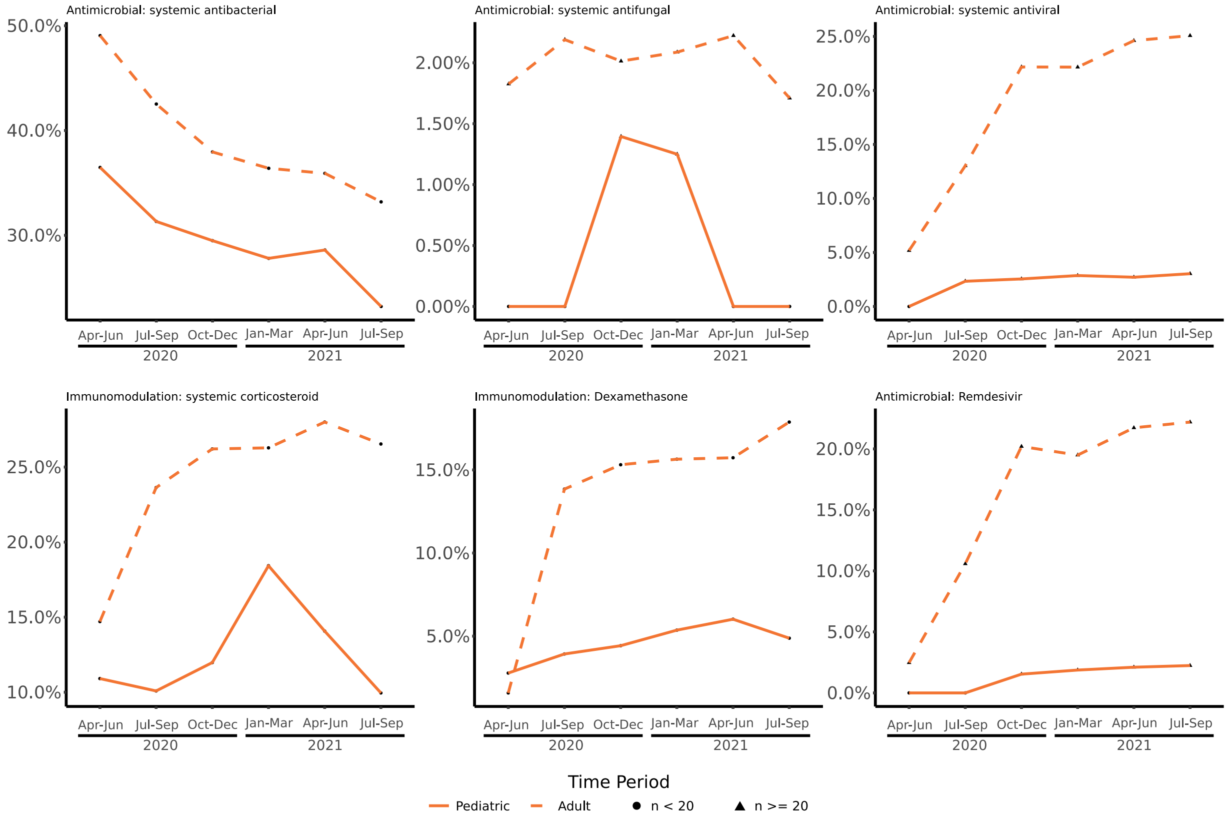

For each three-month period during the study time period, the percent of children and adults who receive a given medication / medication class during their hospitalization is illustrated.

© 2022 Martin B et al. *JAMA Network Open.*

eFigure 6: Additional vital sign and laboratory value in-hospital trajectories

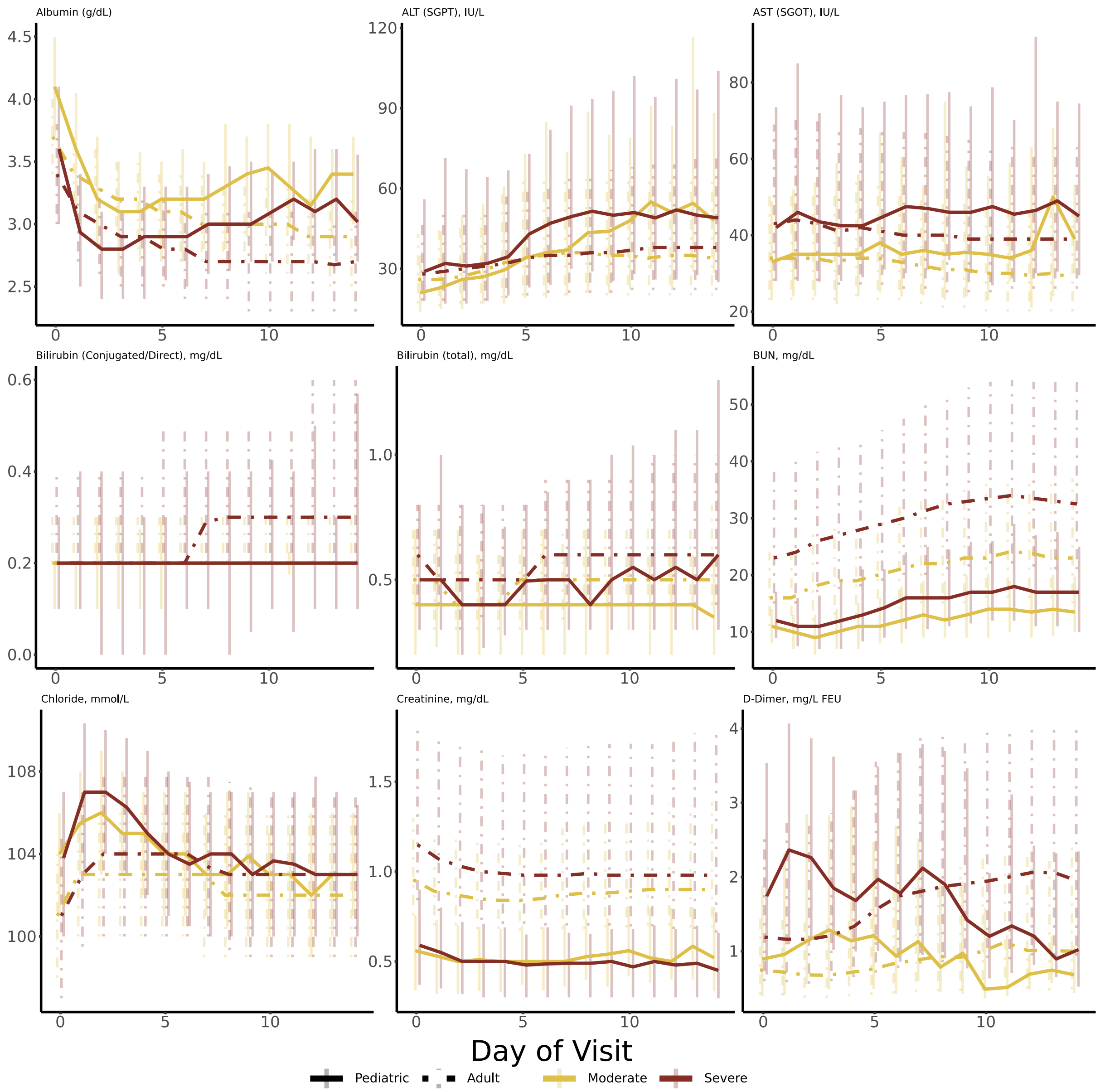

Trajectories of additional selected vital sign and laboratory median values by day of hospitalization during pediatric hospital encounters as compared to N3C adult hospital encounters. For each day of hospitalization, the median and interquartile range (IQR) for those patients with a given vital sign or laboratory value available on that day are calculated. The vertical bars represent the IQR for the values of that specific vital sign or laboratory test value on that day of hospitalization.

© 2022 Martin B et al. *JAMA Network Open.*

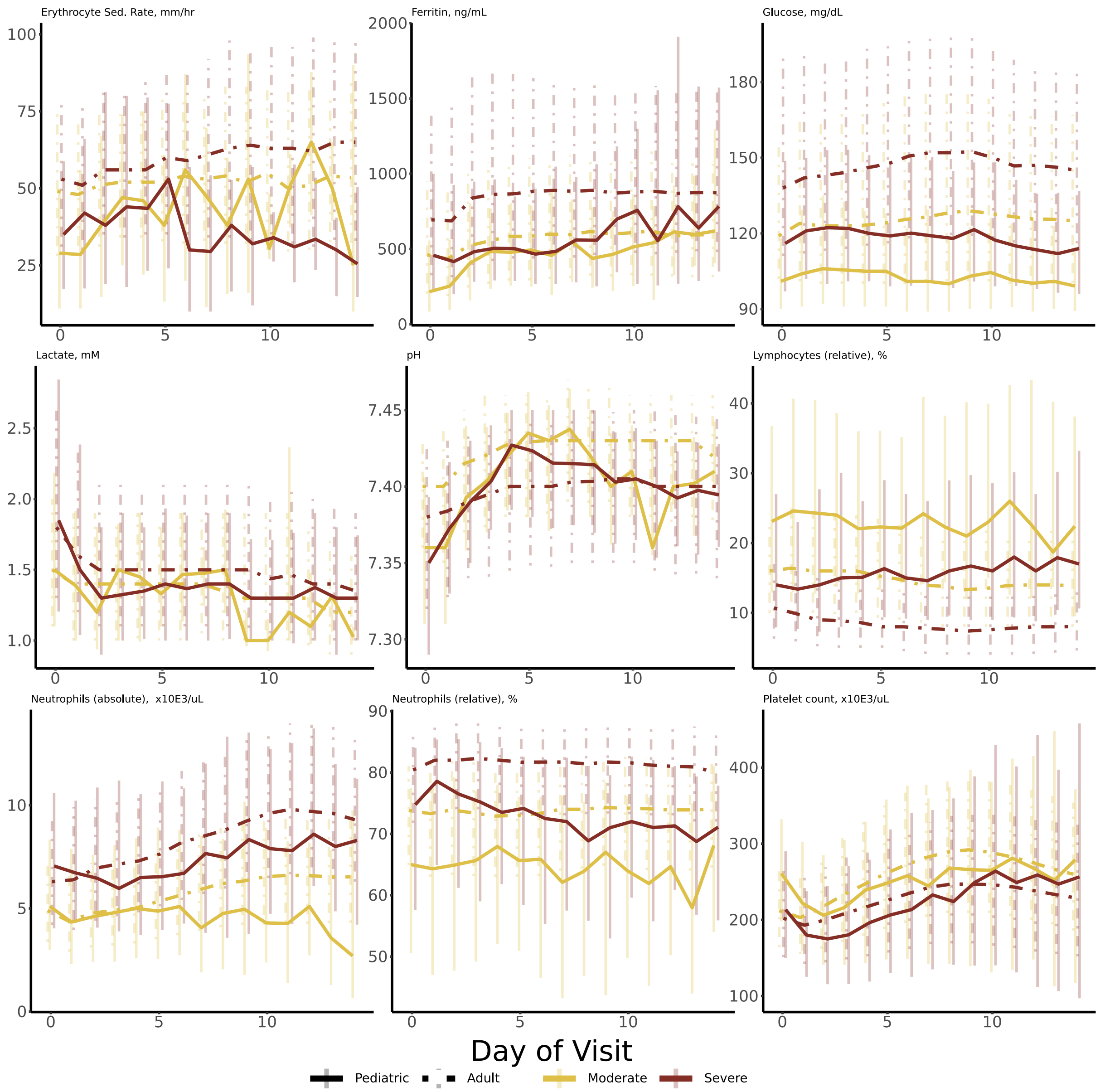

eFigure 6 Continued

© 2022 Martin B et al. *JAMA Network Open.*

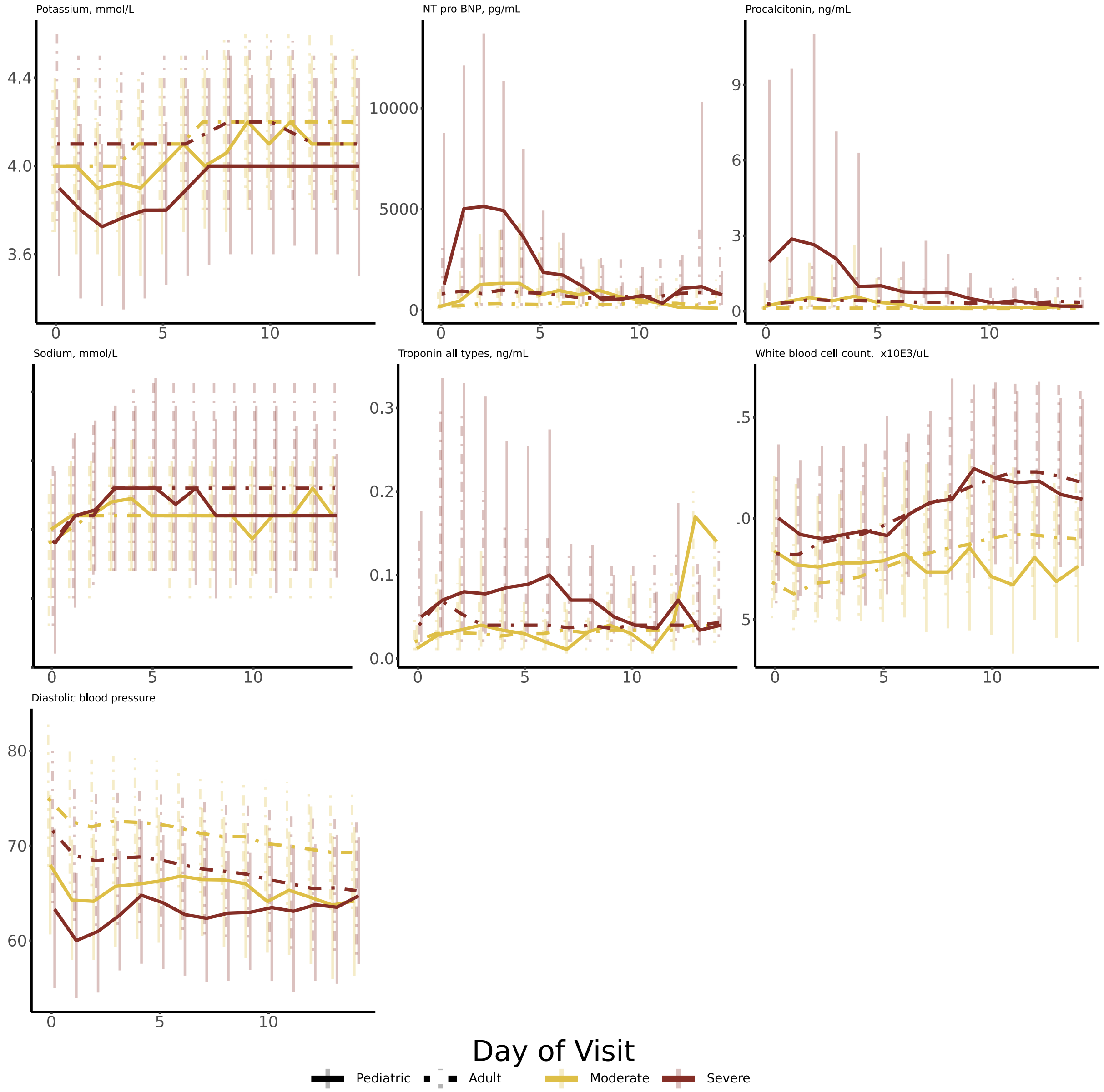

eFigure 6 Continued

© 2022 Martin B et al. *JAMA Network Open.*

eReferences

1. The National COVID Cohort Collaborative. The N3C Phenotype & Data Acquisition Workstream. GitHub. https://github.com/National-COVID-Cohort-Collaborative/Phenotype_Data_Acquisition. Published 2021. Updated March 17, 2021. Accessed June 15, 2021.
2. Bennett TD, Moffitt RA, Hajagos JG, et al. Clinical Characterization and Prediction of Clinical Severity of SARS-CoV-2 Infection Among US Adults Using Data From the US National COVID Cohort Collaborative. *Jama Netw Open.* 2021;4(7):e2116901-e2116901.
3. Haendel MA, Chute CG, Bennett TD, et al. The National COVID Cohort Collaborative (N3C): Rationale, design, infrastructure, and deployment. *J Am Med Inform Assoc.* 2021;28(3):427-443.
4. OHDSI. OMOP Common Data Model. Observational Health Data Sciences and Informatics. https://www.ohdsi.org/data-standardization/the-common-data-model/. Published 2021. Accessed June 18, 2021.
5. Marshall J, Murthy S, Diaz J, Adhikari N, Angus D, Arabi Y. WHO Working Group on the Clinical Characterisation and Management of COVID-19 infection: A minimal common outcome measure set for COVID-19 clinical research. *Lancet Infect Dis.* 2020;20:e192-e197.
6. Feudtner C, Christakis DA, Connell FA. Pediatric deaths attributable to complex chronic conditions: a population-based study of Washington State, 1980-1997. *Pediatrics.* 2000;106(1 Pt 2):205-209.
7. Feinstein JA, Russell S, DeWitt PE, Feudtner C, Dai D, Bennett TD. R package for pediatric complex chronic condition classification. *JAMA pediatrics.* 2018;172(6):596-598.
8. Venkataraman S, Yang Z, Liu D, et al. Sparkr: Scaling r programs with spark. Paper presented at: Proceedings of the 2016 International Conference on Management of Data2016.
9. PySpark Documentation. Apache Spark. http://spark.apache.org/docs/latest/api/python/. Accessed September 1, 2021.
10. Zaharia M, Xin RS, Wendell P, et al. Apache spark: a unified engine for big data processing. *Communications of the ACM.* 2016;59(11):56-65.
11. Wickham H, Chang W, Wickham MH. Package ‘ggplot2’. *Create Elegant Data Visualisations Using the Grammar of Graphics Version.* 2016;2(1):1-189.
12. von Elm E, Altman DG, Egger M, et al. The Strengthening the Reporting of Observational Studies in Epidemiology (STROBE) statement: guidelines for reporting observational studies. *J Clin Epidemiol.* 2008;61(4):344-349.
13. Centers for Disease Control and Prevention. Defining Childhood Obesity. https://www.cdc.gov/obesity/childhood/defining.html. Updated July 3, 2018. Accessed March 1, 2021.
14. Centers for Disease Control and Prevention. Variant Proportions. CDC. Published 2021. Accessed Sep 23, 2021.

© 2022 Martin B et al. *JAMA Network Open.*
